# Lateral Acquisitions Repeatedly Remodel the Oxygen Detoxification Pathway in Diplomonads and Relatives

**DOI:** 10.1093/gbe/evz188

**Published:** 2019-09-05

**Authors:** Alejandro Jiménez-González, Feifei Xu, Jan O Andersson

**Affiliations:** 1 Uppsala Biomedicine Centre, Department of Cell and Molecular Biology, Molecular Evolution Program, Uppsala University, Sweden; 2 Uppsala Biomedicine Centre, Department of Cell and Molecular Biology, Microbiology Program, Uppsala University, Sweden

**Keywords:** protists, parasites, reactive oxygen species, horizontal gene transfer, LGT, HGT

## Abstract

Oxygen and reactive oxygen species (ROS) are important stress factors for cells because they can oxidize many large molecules. Fornicata, a group of flagellated protists that includes diplomonads, have anaerobic metabolism but are still able to tolerate fluctuating levels of oxygen. We identified 25 protein families putatively involved in detoxification of oxygen and ROS in this group using a bioinformatics approach and propose how these interact in an oxygen detoxification pathway. These protein families were divided into a central oxygen detoxification pathway and accessory pathways for the synthesis of nonprotein thiols. We then used a phylogenetic approach to investigate the evolutionary origin of the components of this putative pathway in *Diplomonadida* and other Fornicata species. Our analyses suggested that the diplomonad ancestor was adapted to low-oxygen levels, was able to reduce O_2_ to H_2_O in a manner similar to extant diplomonads, and was able to synthesize glutathione and l-cysteine. Several genes involved in the pathway have complex evolutionary histories and have apparently been repeatedly acquired through lateral gene transfer and subsequently lost. At least seven genes were acquired independently in different Fornicata lineages, leading to evolutionary convergences. It is likely that acquiring these oxygen detoxification proteins helped anaerobic organisms (like the parasitic *Giardia intestinalis*) adapt to low-oxygen environments (such as the digestive tract of aerobic hosts).

## Introduction

Oxygen (O_2_) and reactive oxygen species (ROS) like superoxide ($O_{2}^{\text{–}}$), hydrogen peroxide (H_2_O_2_), and hydroxyl radicals (HO^•^) are important stress factors for both aerobic and anaerobic organisms (Cadenas 1989) because they are highly reactive with macromolecules. For example, ROS can modify proteins via oxidation of cysteine or methionine residues, and they can deactivate metalloenzymes by oxidizing the metal cofactor (especially Fe^2+^). ROS can oxidize lipids by a sequential process called lipid peroxidation, which can result in membrane alterations (Bland 1978). ROS can also damage DNA by creating point mutations and potentially blocking replication (Adelman et al. 1988; Demple and Harrison 1994). Eukaryotes have a variety of enzymatic defenses against ROS, including superoxide dismutase (SOD), catalases, and peroxidases, which reduce $O_{2}^{\text{–}}$ and H_2_O_2_ to H_2_O (Cadenas 1989).

A diversity of such defense mechanisms can be found within Fornicata (Metamonada, Excavata), a group of flagellated protists that live in oxygen-poor environments. Although some members of this group have SOD and peroxidases, others lack all three main players in oxidative stress response (Brown et al. 1995, 1998; Raj et al. 2014; Xu et al. 2014). For instance, the diplomonad *Giardia intestinalis* uses an A-type flavoprotein, an NADH oxidase, and a flavohemoprotein as its primary defenses against ROS (Vicente et al. 2009; Testa, Giuffrè, et al. 2011; Ma’ayeh et al. 2015). *Giardiaintestinalis* also uses l-cysteine as an important nonprotein thiol antioxidant (Brown et al. 1993). l-Cysteine occurs either as a free amino acid or as a precursor to glutathione (Krauth-Siegel and Leroux 2012), which reduces HO^•^ and lipid radicals to H_2_O and stable lipids, respectively, or acts as a cofactor of several enzymes.

Fornicata lacks classical mitochondria and instead has a fermentative metabolism in which glucose is reduced to pyruvate via glycolysis (Lindmark 1980; Müller 1988). Pyruvate is then decarboxylated to acetyl-CoA by the enzyme pyruvate:ferredoxin oxidoreductase (PFO), and the electron released by PFO is transported via ferredoxin to produce NADH. Both PFO and ferredoxin are oxygen labile, which means that ROS can interfere with or entirely stop this metabolic pathway (Townson et al. 1994; Brown et al. 1998). In principle, therefore, diplomonads and other Fornicata species should be especially vulnerable to ROS. However, instead, these organisms tolerate relatively high levels of oxygen and can be found living in anaerobic or low-oxygen cavities within aerobic species (Espey 2013; Williams et al. 2014; Ma’ayeh et al. 2015; Stairs et al. 2019). Clearly diplomonads—and most likely other members of Fornicata—have evolved efficient mechanisms for dealing with oxidative stress in their normal environment, or from accidental exposure to oxygen at different stages of their life cycles, despite lacking SOD and peroxidases.

The genomes and transcriptomes for a number of species in Fornicata (both free living and host associated) have been published recently (Xu et al. 2014, 2016; Leger et al. 2017; Tanifuji et al. 2018), allowing us to use a comparative phylogenetic approach to learn more about the evolution of this detoxification pathway. In this article, we reconstruct a putative oxygen detoxification pathway for Fornicata species based on comparative genomics and analyze the origins of the different parts of this pathway using a phylogenetic approach.

## Materials and Methods

### Data Sources

Protein sequences from the genome data sets of the diplomonads *G.intestinalis* WB, *G. intestinalis* DH, *G. intestinalis* GS, *G. intestinalis* GS_B, *G. intestinalis* P15, and *Spironucleus salmonicida* (Metamonada: Fornicata) as well as the distantly related *Trichomonas vaginalis* (Metamonada: Parabasalia) were downloaded from EuPathDB (Aurrecoechea et al. 2013) and NCBI (NCBI Resource Coordinators 2017). Protein sequences from the genome of *Monocercomonoides exilis* (Metamonada: Preaxostyla; Karnkowska et al. 2016) were downloaded from protistologie.cz/hampllab/data.html. Protein sequences from the transcriptome data set of the free-living diplomonad *Trepomonas* sp. PC1 (Metamonada: Fornicata; Xu et al. 2016) and the genome data set of the free-living nondiplomonad *Kipferlia bialata* (Metamonada: Fornicata; Tanifuji et al. 2018) were downloaded from NCBI. Nucleotide sequences from the transcriptome data sets of the nondiplomonad Fornicata species *Aduncisulcus paluster*, *Carpediemonas membranifera*, *Chilomastix caulleryi*, *Chilomastix cuspidata*, *Dysnectes brevis*, and *Ergobibamus cyprinoides* and the Preaxostyla *Trimastix marina* (Leger et al. 2017) were downloaded from Dryad Digital Repository. Sequences from *Giardia muris* were acquired from an ongoing genome project (Xu F, Jiménez-González A, Einarsson E, Ástvaldsson A. Peirasmaki D, Eckmann L, Andersson JO, Svärd SG, Jerlström-Hultqvist J., unpublished data).

### Reconstruction of the Oxygen Detoxification Pathway and Identification of Candidate Proteins

The putative oxygen detoxification pathway in *G. intestinalis* and *S. salmonicida* was reconstructed in two steps (supplementary fig. 1, Supplementary Material online). First, homologs of proteins involved in ROS detoxification in *G. intestinalis*, *S. salmonicida*, and/or *T. vaginalis* (Kurtz 2006; Westrop et al. 2006; Di Matteo et al. 2008; Mastronicola et al. 2010; Testa, Mastronicola, et al. 2011; Krauth-Siegel and Leroux 2012; Xu et al. 2014; Ma’ayeh et al. 2015; Castillo-Villanueva et al. 2016; Stairs et al. 2019) were identified in the sequenced diplomonads *G. intestinalis* and *S. salmonicida*. Second, proteins that have previously been identified as involved in oxygen detoxification in other species (Almeida et al. 2006; Delaye et al. 2007; Andisi et al. 2012; Krauth-Siegel and Leroux 2012; Cabeza et al. 2015; Beltrame-Botelho et al. 2016) were used as queries in BlastP searches against *G. intestinalis* and *S. salmonicida* genomes. The protein domains of each putative detoxification protein were evaluated using the Conserved Domain database (Marchler-Bauer and Bryant 2004), and, if these appeared to be correct, the protein was added to the pathway.

These proteins were then used as BlastP and PSI-BLAST queries against the genomes of *G. muris*, *K. bialata*, and *M*. *exilis*, and recovered candidate sequences were evaluated as described above. Homologs of oxygen detoxification proteins (Almeida et al. 2006; Delaye et al. 2007; Andisi et al. 2012; Krauth-Siegel and Leroux 2012; Cabeza et al. 2015; Beltrame-Botelho et al. 2016) which were absent in *G. intestinalis* and *S. salmonicida* were identified in these genomic data sets, evaluated, and added to the pathway.

**Fig. 1.**
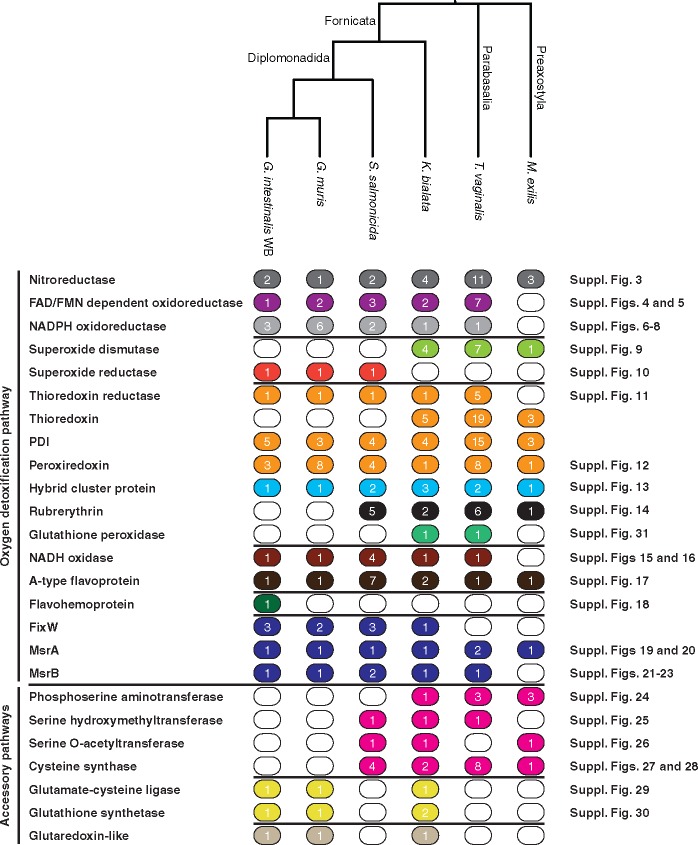
—Distribution of proteins across Metamonada. Filled ovals denote presence of the protein, whereas empty circles denote absence. The numbers inside the circles indicate the number of copies within respective genomes. PDI, protein disulfide isomerase; MsrA, methionine sulfoxide reductase A; MsrB, methionine sulfoxide reductase B.

Finally, TBlastN searches against the downloaded transcriptomes named earlier were performed, using as queries only proteins confirmed in at least one Fornicata genome. The obtained hits were translated into amino acid sequences using EMBOSS Sixpack (Madeira et al. 2019) and evaluated using the Conserved Domain database (Marchler-Bauer and Bryant 2004). The result of these procedures was that we created curated Metamonada databases of 25 proteins families involved in oxygen detoxification in Fornicata (fig. 1 and supplementary fig. 1, Supplementary Material online).

### Data Matrix Assembly

We could not include all available sequences of the 25 proteins families because it is simply not feasible to perform phylogenetic analyses of thousands of sequences with the currently available software and hardware. Therefore, our goal was to assemble phylogenetic matrices with a restricted number of sequences that would still be diverse enough to reliably identify the origin of query sequences. To evaluate how many sequences were needed, we used the following procedure.

For each of the 25 proteins, when possible, a sequence from either *G. intestinalis* or *S. salmonicida* and a sequence from *K. bialata* were used as queries against NCBI nr database (June 2018). For *G. intestinalis* proteins with multiple copies, the copy most highly expressed under oxygen stress conditions was used as a query (Ma’ayeh et al. 2015). Each of these BlastP searches was repeated with three different settings, keeping 1,000, 5,000, or 10,000 hits with *e*-values <0.001. For each protein, the matrices based on the *G. intestinalis* or *S. salmonicida* and *K. bialata* searches were combined, and the results were filtered using CD-HIT (Li and Godzik 2006) by keeping only sequences with <90% sequence identity to another sequence in the data set (supplementary fig. 2, Supplementary Material online). These filtered matrices were then merged with homologous proteins from the curated Metamonada databases that we had created previously (see above, supplementary fig. 1, Supplementary Material online) and aligned using MAFFT v6.603b (Katoh et al. 2002) with the default settings. The resulting alignments were trimmed using BMGE v1.12 (BLOSUM30 with a block size of 2, Criscuolo and Gribaldo 2010).

For each protein matrix, preliminary phylogenetic trees were computed using FastTree v2.1.8 SSE3, OpenMP (Price et al. 2010) with the default settings. The smaller matrix was selected if the major groups present in the larger matrices were already represented, and the diversity phylogenetically close to the query sequences was highly similar in the three matrices. The optimal number of hits to include from the BlastP searches was 1,000 for all the proteins, except for hybrid-cluster protein and serine hydroxymethyltransferase for which the optimal number was 10,000 (supplementary fig. 2, Supplementary Material online).

The fraction of hits in common between the two selected BlastP searches (*G. intestinalis* or *S. salmonicida* and *K. bialata*) was calculated using CD-HIT-2D (Li and Godzik 2006) with the default settings. If the fraction of common hits was >20%, the diversity matrix was used in the final trimming steps, independent of the result of the preliminary tree. If *K. bialata* and diplomonad sequences did not cluster together in the preliminary tree and the fraction of common hits was <20%, the diversity matrix was split and the proteins were considered to have independent evolutionary histories (supplementary table 1, Supplementary Material online).

Occasionally, diplomonad genomic sequences clustered independently to the diplomonad query sequence in the initial trees. In such cases, new BlastP searches were run using representative sequences from this independent diplomonad cluster. Pairwise fractions of hits in common between the two different BlastP searches for the protein were calculated and the matrices were merged or split according to the rules outlined above. After every split, the BlastP-generated matrices were reused and new rounds of CD-HIT were performed as described above. The new matrices were merged with the relevant sequences from the curated Metamonada database (supplementary fig. 2, Supplementary Material online).

A new FastTree was computed as described above after the split and merge process. Sequences with a phylogenetic distance <0.3 were removed in an iterative process to further reduce the size of the matrix, until the final matrix was generated.

### Phylogenetic Analysis

The final matrices were aligned using MAFFT and trimmed using BMGE as described above. Maximum likelihood trees were computed using IQtree v1.5.3 (Nguyen et al. 2015) under LGX substitution model. Branch supports were assessed using ultrafast bootstrap approximation (UFboot) with 1,000 bootstrap replicates (Hoang et al. 2018) and SH-like approximative likelihood ratio test (SH-aLRT) (Guindon et al. 2010), for which 1,000 replicates were used.

To avoid the inclusion of sequences from contamination, the genomic contexts and the presence of intron were inspected for Fornicata sequences that branch independently. Introns were present in all inspected *K. bialata* genes and all inspected diplomonads genes were located on large eukaryotic contigs. Sequences from Fornicata transcriptomes were removed from matrices if they did not cluster with any Fornicata species with a sequenced genome. After every pruning, a new IQtree was computed. These steps were repeated until all suspected contaminants from the transcriptomes had been eliminated, and final phylogenetic trees were then computed.

Four trees (FAD/FMN-dependent oxidoreductase group 1, GSH1, GSH2, and peroxiredoxin) had long branches for some Fornicata species. To reduce long-branch attraction artifacts, additional phylogenetic trees were computed keeping only the taxa with the shortest Fornicata branches. Both trees are included in the supplementary material, Supplementary Material online.

### Phylogenetic Trees Interpretation

We considered UFboot ≥ 95% and SH-aLRT ≥ 80% for bipartitions as good support following IQtree manual. A protein family was considered ancestral in Fornicata when it clustered with eukaryotic sequences from all eukaryotic supergroups with good support. If a protein or protein family clustered with prokaryotic sequences, other than *Alphaproteobacteria*, with good support, that protein was considered potentially acquired from prokaryotes via lateral gene transfer (LGT). If a tree had low support but the matrix was composed primarily of prokaryotic and Fornicata sequences, then that protein family was considered potentially acquired from prokaryotes via LGT. Finally, a Fornicata protein was considered potentially acquired if it clustered with sequences from distant eukaryotic lineages which in turn clustered with prokaryotes. The phylogenetic distribution of the protein was also taken into consideration when interpreting its evolutionary origin. See supplementary table 2, Supplementary Material online, for details of how these rules were applied to the proteins analyzed with phylogenetic methods.

## Results

**Fig. 2.**
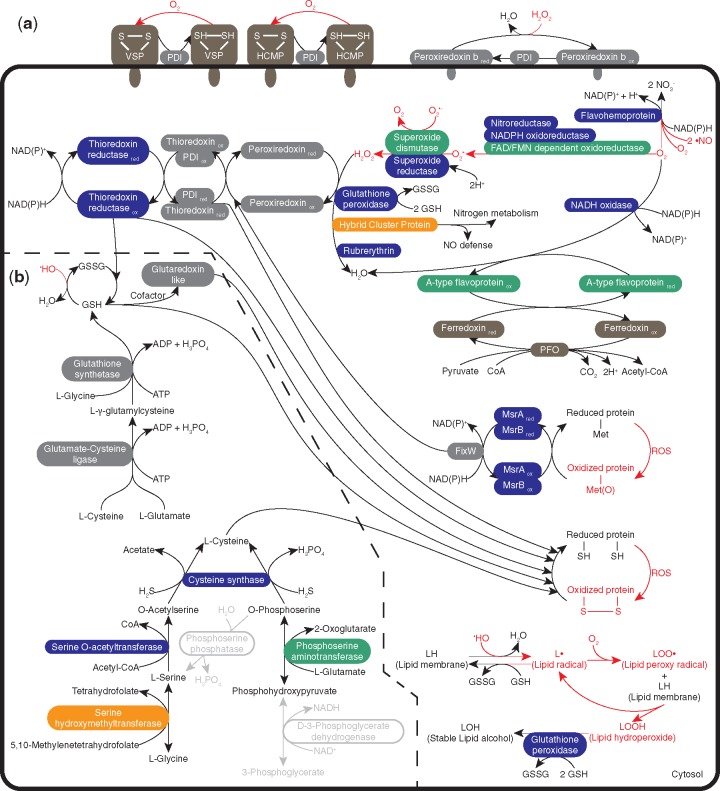
—Oxygen detoxification pathway combining all the analyzed fornicata species. (*a*) Oxygen detoxification pathway and (*b*) Accessory pathways. Proteins inferred to be ancestral are in orange. Proteins likely to have been acquired before the last Fornicata common ancestor are in green. Proteins likely to have been acquired after the last Fornicata common ancestor are in dark blue. Proteins with an unclear origin are in gray. Enzyme reactions absent from Fornicata are in light gray. Proteins that might play a role in the detoxification pathway but were not included in the analysis are in brown. Reactions with more than one protein do not indicate that those proteins interact. Toxic reactions and toxic molecules for the cell are in red. ROS, reactive oxygen species; PDI, protein disulfide isomerase; MsrA, methionine sulfoxide reductase A; MsrB, methionine sulfoxide reductase B; GSH, glutathione; GSSH, glutathione disulfide; VSP, variable surface protein; HCMP, high cysteine membrane protein; PFO, pyruvate:ferredoxin oxidoreductase; ox, oxidized conformation of the protein; red, reduced conformation of the protein.

Our initial search for genes pertaining to the O_2_-scavenging system in published Fornicata genomes and transcriptomes and the unpublished *G. muris* genome identified 25 proteins as potentially contributing to defense against oxygen (fig. 1). We divided these proteins into two main groups: proteins forming an oxygen detoxification pathway (fig. 2*a*) and proteins in accessory pathways involved in the synthesis of nonprotein thiols (fig. 2*b*). The oxygen detoxification pathway included direct systems that reduce ROS to H_2_O, and indirect systems that repair protein and membrane damage. Twelve of the 25 protein families were present in all available Fornicata genomes (fig. 1). Six of these 12 protein families were also present in *T. vaginalis* and *M. exilis* (distantly related members of Metamonada). This distribution suggests that part of the detoxification pathway might be ancestrally present, whereas other parts might be lineage specific with gains and losses of different parts of the pathway through adaptation to microaerophilic environments.

Our phylogenetic testing of the proteins resulted in 29 trees (fig. 3 and supplementary figs. 3–31, Supplementary Material online). The trees showed that two proteins involved in the oxygen detoxification pathway in Fornicata were ancestrally present, three proteins were present in the last Fornicata common ancestor, but with an unclear origin and 16 were acquired by at least one lineage in Fornicata after the diversification of the eukaryotic supergroups. Four proteins (protein disulfide isomerase [PDI], glutaredoxin-like, thioredoxin, and FixW, all members of the thioredoxin-like superfamily) failed to produce reliable alignments and were excluded from evolutionary analyses. The timing of the acquisitions was determined using the information from the phylogenetic distribution in combination with the results from the phylogenetic analyses. This revealed a complex evolutionary history of the oxygen detoxification proteins in Fornicata (fig. 4).

**Fig. 3.**
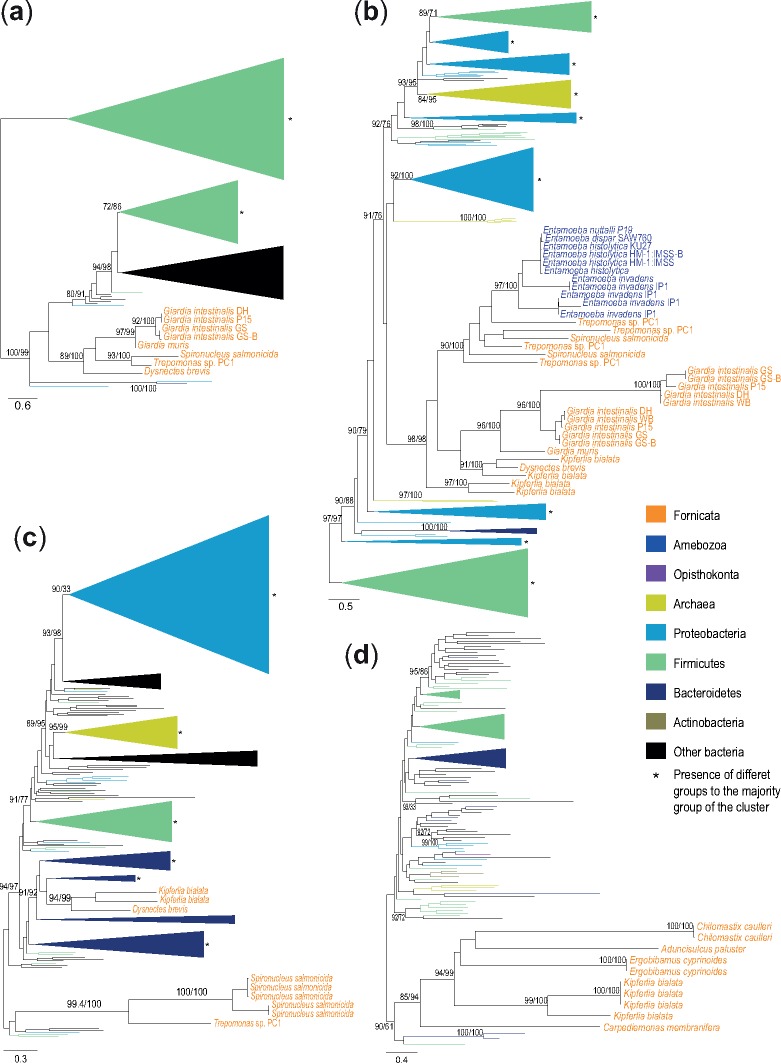
—Phylogenetic trees of four protein families involved in the oxygen detoxification pathway. (*a*) SOR, (*b*) nitroreductase, (*c*) rubrerythrin, and (*d*) SOD. Maximum likelihood topologies with key ultrafast bootstrap support values followed by SH-aLRT bootstrap support values. Complete phylogenetic trees of these protein families are found in supplementary figures 10, 3, 14, and 9, Supplementary Material online, respectively.

**Fig. 4.**
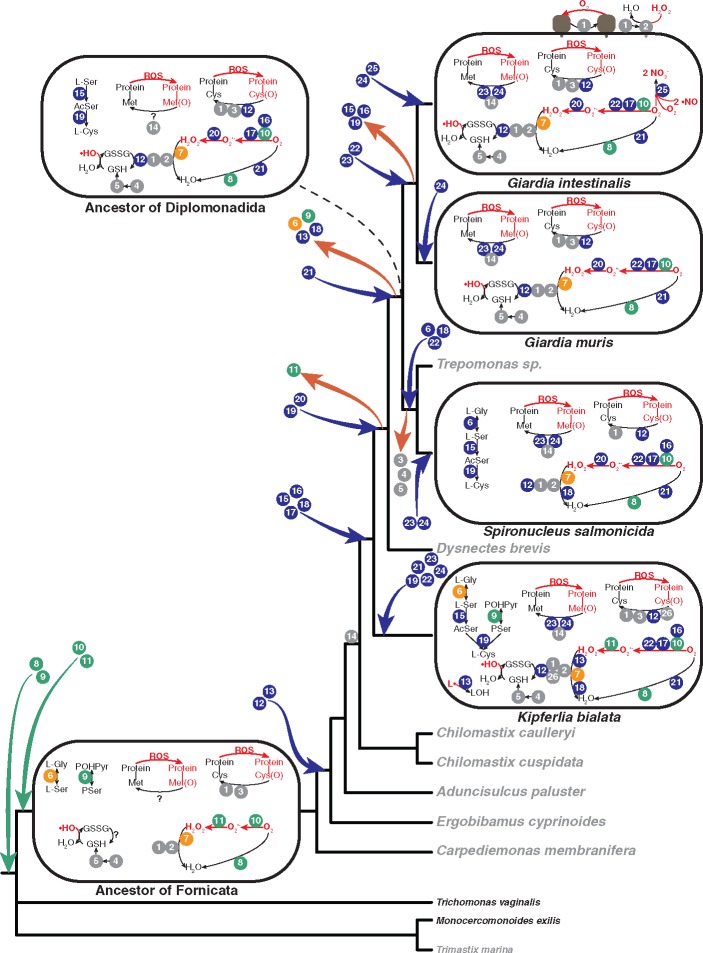
—Evolutionary transitions of the oxygen detoxification pathway from the last Fornicata common ancestor to extant Fornicata species. Proteins inferred to be ancestral are in orange. Proteins likely to have been acquired before the last Fornicata common ancestor are in green. Proteins likely to have been acquired after the last Fornicata common ancestor are in dark blue. Proteins with an unclear origin are in gray. Variable surface protein and HCMP in *Giardia intestinalis* are in brown. Species with genomic and transcriptomic data are in black and gray, respectively. Cys(O), nonnative oxidized cysteine; Met(O), nonnative oxidized methionine; GSH, glutathione; GSSH, glutathione disulfide; l-Ser, serine; l-Gly, glycine; l-Cys, cysteine; POHpyr; phosphohydroxypyruvate; PSer, *O*-phosphoserine; AcSer, *O*-acetylserine. The numbers indicate the identity of the protein: 1, PDI; 2, peroxiredoxin; 3, glutaredoxin-like protein; 4, glutamate-cysteine synthase; 5, glutathione synthase; 6, serine hydroxymethyltransferase; 7, hybrid-cluster protein; 8, A-type flavoprotein; 9, phosphoserine aminotransferase; 10, FAD/FMN-dependent oxidoreductase group 1; 11, SOD; 12, thioredoxin reductase; 13, glutathione peroxidase; 14, fixW; 15, serine *O*-acetyltransferase; 16, FAD/FMN-dependent oxidoreductase group 2; 17; nitroreductase; 18, rubrerythrin; 19, cysteine synthase; 20, SOR; 21, NADH oxidase; 22, NADPH oxidoreductase; 23, MsrA; 24, MsrB; 25; flavohemoprotein; 26; thioredoxin.

### Reduction of Oxygen to Water via Superoxide Radicals and Hydrogen Peroxide

In our proposed pathway, oxygen can be reduced to H_2_O by two different routes in the cell (fig. 2*a*), the first of which involves three steps via superoxide radicals ($O_{2}^{\text{•–}}$) and hydrogen peroxide (H_2_O_2_).

#### Step 1: Reduction of Oxygen to Superoxide Radical

Fornicata species encode at least three different enzymes that can produce $O_{2}^{\text{•–}}$, namely nitroreductase, FAD/FMN-dependent oxidoreductase, and NADPH oxidoreductase (fig. 2*a*). The exact roles of these proteins are still unclear, but they are overexpressed under ROS stress conditions (Ma’ayeh et al. 2015; Stairs et al. 2019) and produce a molecule of $O_{2}^{\text{•–}}$ when O_2_ is the electron acceptor (Li and Wang 2006; Macheroux et al. 2011).

The first enzyme, nitroreductase, appeared to have a common origin in *K. bialata* and diplomonads through gene transfer from bacteria (fig. 3b and supplementary fig. 3, Supplementary Material online). The phylogeny of nitroreductase also suggested a secondary eukaryote-to-eukaryote lateral transfer of nitroreductase from diplomonads to the *Entamoeba* lineage.

The second enzyme, FAD/FMN-dependent oxidoreductase, has two different types in all Fornicata genomes except *Giardia*, which has only one type. Our analyses suggested that these two types originated from two independent LGT events. The first type was sister group to Opisthokonta, and Fornicata + Opisthokonta cluster with bacteria, supporting a bacterial origin of the eukaryotic genes. There are two plausible alternative scenarios for the distribution of the gene within eukaryotes. Either the gene was acquired once early in eukaryotic evolution and subsequently lost in multiple lineages independently, or the gene was transferred from bacteria to a member of Opisthokonta or Fornicata, and later transferred between groups (supplementary fig. 4*a* and *b*, Supplementary Material online). We tend to favor the latter scenario because of the patchy phylogenetic distribution of homologs of FAD/FMN-dependent oxidoreductase within eukaryotes. The second type of FAD/FMN-dependent oxidoreductase was acquired before the split of *K. bialata* from the lineage leading to diplomonads and was then subsequently lost in *Giardia* (supplementary fig. 5, Supplementary Material online). *Trichomonasvaginalis* homologs of this protein formed two independent clusters. In total, then, our topologies suggest that this second type of FAD/FMN-dependent oxidoreductase had three independent origins in Fornicata and *T. vaginalis*.

The third enzymes, NADPH oxidoreductases, were only found in diplomonads and *K. bialata* genomes (fig. 1). The lack of overlap between the data matrices suggested independent evolutionary histories for this protein in *Giardia*, *S. salmonicida*, and *K. bialata* genomes (supplementary table 1, Supplementary Material online). The phylogenetic trees showed a bacterial origin for all three homologs but do not allow us to pinpoint specific donor groups (supplementary figs. 6–8, Supplementary Material online).

#### Step 2: Reduction of Superoxide Radicals to Hydrogen Peroxide

The second step in this route of the detoxification of ROS to water is the reduction of $O_{2}^{\text{•–}}$ to H_2_O_2_. In our proposed pathway, this reaction can be carried out by SOD or superoxide reductase (SOR) (fig. 2*a*). Here, we observed a clear division in distribution within Fornicata. Although SOR was present only in diplomonads, homologs of SOD were found in all other genomes analyzed (fig. 1).

Interestingly, Fornicata SOD seems to have been acquired by LGT from bacteria (fig. 3*d* and supplementary fig. 9, Supplementary Material online). The final data set of SOR included only Fornicata and prokaryotic sequences (fig. 3*a* and supplementary fig. 10, Supplementary Material online). Fornicata sequences branched with a diversity of bacteria sequences in the SOD and SOR trees, making it impossible to pinpoint donor lineages. Nevertheless, our analysis showed that SOD was replaced by SOR before the split between *Dysnectes brevis* and diplomonads (fig. 4).

#### Step 3: Reduction of Hydrogen Peroxide to Water

H_2_O_2_ is reduced to H_2_O in the final step of this detoxification route (fig. 2*a*). For this step, catalases, peroxidases, and peroxiredoxins are widespread in eukaryotes, but Fornicata genomes did not encode catalases and only *K. bialata* encoded a glutathione peroxidase (fig. 1). Fornicata species use a peroxiredoxin complex formed by three enzymes that transfer electrons from NAD(P)H to H_2_O_2_ (fig. 2*a*). The first step is driven by the thioredoxin reductase. This protein takes electrons from NAD(P)H and transfers them to thioredoxin. Thioredoxin then transfers these electrons to a peroxiredoxin protein that catalyzes the reduction of H_2_O_2_ to H_2_O (Mastronicola et al. 2014). Homologs of thioredoxin have been found in *K. bialata*, but our searches for thioredoxin homologs in diplomonad proteomes returned only PDIs, even though some proteins have been annotated as thioredoxin-like protein (Morrison et al. 2007). The absence of a functional thioredoxin has been reported previously in *G. intestinalis* (Leitsch et al. 2017). PDI proteins belong to the same protein family as thioredoxin and share the role of rearranging nonnative disulfide bonds, and it is possible that PDI might serve the function of thioredoxin in diplomonads.

One peroxiredoxin and PDI paralog in *G. intestinalis* is secreted or exposed on the outside of the membrane (Mastronicola et al. 2014; Ma’ayeh et al. 2017). This suggests the existence of a partial peroxiredoxin complex working as the first line of defense against ROS (fig. 2*a*). *Giardiaintestinalis* expresses on the outside membrane two types of cysteine-rich proteins, variant surface protein and high cysteine membrane protein (HCMP) (Davids et al. 2006). We propose that the PDI paralog secreted or exposed on the outside membrane is responsible for restoring the damage caused by O_2_ and ROS in these proteins (fig. 2*a*).

Our analyses suggested that thioredoxin reductase was acquired after the split between *Carpediemonasmembranifera* and the rest of Fornicata (supplementary fig. 11, Supplementary Material online). Fornicata sequences clustered within a heterogeneous group composed of bacterial and eukaryotic sequences, separate from the main eukaryotic group (supplementary fig. 11, Supplementary Material online). The peroxiredoxin phylogenetic tree showed that this protein was present in the last Fornicata ancestor. Our analyses suggested that there had been one eukaryote-to-eukaryote LGT event between Opisthokonta and Fornicata, but the direction of this event could not be resolved (supplementary fig. 12*a* and *b*, Supplementary Material online), nor could our analysis solve the evolutionary history of PDI and thioredoxin.

There are three additional enzymes for the reduction of H_2_O_2_ to H_2_O in Fornicata, namely hybrid-cluster protein (Almeida et al. 2006), rubrerythrin (Kurtz 2006), and glutathione peroxidase (Mannervik 1985) (fig. 2*a*). The first enzyme, hybrid-cluster protein, plays a dual role of both H_2_O_2_ detoxification and nitrogen metabolism (Schneider et al. 2011). The hybrid-cluster protein phylogeny resolved three independent eukaryotic clusters (supplementary fig. 13, Supplementary Material online): The *Entamoeba* sequences clustered inside a proteobacteria group, two *Trichomonas* sequences clustered together within a diverse bacterial group, and *M*. *exilis*, *Trimastixmarina*, and Fornicata sequences clustered together within a diverse eukaryotic group with moderate support. Hybrid-cluster protein was therefore considered ancestral to Fornicata.

The second enzyme, rubrerythrin, was present in all included genomes except *G. intestinalis* and *G. muris* (fig. 1). Phylogenetic analysis suggested that homologs of this gene were acquired independently (fig. 3*c* and supplementary fig. 14, Supplementary Material online) by *D. brevis* and *K. bialata*, and *S. salmonicida* and *Trepomonas* sp. PC1. Our analyses also suggested that this protein was lost in the last *Diplomonadida* common ancestor and then reacquired in the lineage leading to *S. salmonicida* and *Trepomonas* sp. PC1 (fig. 4).

### Reduction of Oxygen Directly to Water

Diplomonads can also reduce oxygen directly to H_2_O by two different proteins, NADH oxidase (Brown et al. 1996a) and A-type flavoprotein (Di Matteo et al. 2008). Homologs of these proteins were found in all analyzed species. NADH oxidases reduce O_2_ to H_2_O_2_ and/or H_2_O. In diplomonads, this enzyme reduces O_2_ directly to H_2_O without producing any other ROS (Brown et al. 1996a). Flavoproteins reduce either O_2_ or NO. In *G. intestinalis*, A-type flavoprotein has a preference for O_2_, showing a low NO reduction activity (Di Matteo et al. 2008). In the parasites *T. vaginalis* and *E. histolytica*, oxidized A-type flavoprotein can be reduced via the PFO/ferredoxin system (Smutná et al. 2009; Cabeza et al. 2015). Both PFO and ferredoxin are upregulated under oxygen stress conditions in diplomonads (Ma’ayeh et al. 2015; Stairs et al. 2019), which suggests that they are involved in detoxification of ROS. We propose that this system is responsible for the reduction of A-type flavoprotein in Fornicata as well, because we did not detect any other proteins usually described as electron donors (Saraiva et al. 2004) in the different genomes.

The NADH oxidase data matrices of diplomonads and *K. bialata* had no sequences in common, suggesting that they have independent origins (supplementary table 1, Supplementary Material online). *Kipferliabialata* clustered with a diverse set of bacterial sequences (supplementary fig. 15, Supplementary Material online). In diplomonads, the phylogenetic tree supported a single acquisition of the gene from Firmicutes before the diversification of diplomonads, with gene duplications in *S. salmonicida* and *Trepomonas* sp. PC1 (supplementary fig. 16, Supplementary Material online).

A-type flavoprotein was most likely present in the last Fornicata common ancestor because all Metamonada sequences (i.e., all eukaryotes in the tree) clustered together. The most likely cause of this pattern is that this protein is an ancient acquisition from prokaryotes (supplementary fig. 17, Supplementary Material online).

### Flavohemoprotein: •NO and O_2_ Detoxification in *G. intestinalis*

Another protein for detoxification of oxygen, flavohemoprotein, was only found in *G. intestinalis* (fig. 1). Flavohemoproteins oxidize two nitric oxide radicals (•NO) to two nitrates molecules (NO_3_^−^) using two O_2_ (Mastronicola et al. 2010) (fig. 2*a*). Phylogenetic analysis of this gene resolved *G. intestinalis* as sister group to four host-associated members of Trypanosomatidae, and this group itself was sister group to a free-living amoeba (supplementary fig. 18, Supplementary Material online). This group of eukaryotes was sister group to a gammaproteobacterial cluster, suggesting that flavohemoprotein was acquired from bacteria and subsequently transferred among the eukaryotic lineages. However, our analysis could not resolve which lineage was the first LGT recipient, nor the directions of the subsequent transfers.

### Restoring Oxidized Methionine Residues: MsrA and MsrB

An organism cannot avoid all damage caused by ROS, so systems that repair damaged proteins and lipids are needed (fig. 2*a*). Methionine sulfoxide reductase A (MsrA) and methionine sulfoxide reductase B (MsrB), which have independent evolutionary origins (Delaye et al. 2007), both restore oxidized methionine residues. Our analyses showed that different Fornicata lineages acquired both proteins through independent LGT events. In the MsrA phylogenetic tree, *Giardia* clustered with Firmicute sequences (with good support), whereas *S. salmonicida* clustered (with low support) within *Mycoplasma* (supplementary fig. 19, Supplementary Material online). The phylogenetic tree of *K. bialata* showed it clustering within Proteobacteria with low support (supplementary fig. 20, Supplementary Material online).

MsrB has an equally complex evolutionary history within Fornicata. The two diplomonad genera and *K. bialata* had high diversity, resulting in three independent data sets for the phylogenetic analyses (supplementary table 1, Supplementary Material online). *Giardiaintestinalis* sequences clustered with a diverse bacterial group, whereas *G. muris* is sister group to a Thaumarchaeota sequence within a diverse bacterial cluster (supplementary fig. 21, Supplementary Material online). The *K. bialata* homolog clustered (with low support) with proteobacterial sequences (supplementary fig. 22, Supplementary Material online), whereas the *S. salmonicida* MsrB clustered with a *Paramecium* and an Opisthokonta sequence, suggesting two eukaryote-to-eukaryote LGT events. However, our data did not resolve the direction of these events (supplementary fig. 23, Supplementary Material online). Together, these observations suggest that MsrB homologs were acquired four times independently in the *Giardia*, *Spironucleus*, and *Kipferlia* lineages.

MsrA and MsrB need to be recycled after they are used to reduce a damaged protein. The protein thought to be involved in this process in diplomonads and *K. bialata* is FixW, a member of TipA-like family. These proteins act as disulfide reductases that can recycle methionine sulfoxide reductases (Andisi et al. 2012). We propose the same role of the fornicata homologs and, because of its disulfide reductase activity, this protein might also play a role in the peroxiredoxin complex (fig. 2*a*). Our analysis could not resolve the evolutionary history of this protein.

### Restoring Oxidized Cysteine Residues by Low-Molecular-Weight Antioxidants

The oxidation of cysteine residues by ROS induces the creation of nonnative disulfide bonds. There are several mechanisms for restoring these bonds within the cell, both via proteins and low-molecular-weight antioxidants (fig. 2*a* and *b*).

Cysteine is the precursor for all thiol antioxidants, such as glutathione, and is also itself an antioxidant. It is the most important nonprotein thiol in *G. intestinalis*, *S. salmonicida*, and *T. vaginalis* (Brown et al. 1993; Westrop et al. 2006; Xu et al. 2014). There are two different pathways for synthesizing cysteine. One pathway leads to the synthesis of l-cysteine from l-glycine, whereas the other uses 3-phosphoglycerate (fig. 2*b*). Both *S. salmonicida* and *K. bialata* encode genes for the synthesis of cysteine from l-glycine (Xu et al. 2014; Tanifuji et al. 2018). The second pathway, which synthesizes l-cysteine from 3-phosphoglycerate (Westrop et al. 2006), is partially present in *K. bialata* but absent in all analyzed diplomonads. Both pathways are connected by the enzyme phosphoserine phosphatase. However, no gene for this enzyme was found in any of the genomes. *Giardia* genomes lack all genes for both pathways (fig. 1).

Phosphoserine aminotransferase is the only enzyme detected for the synthesis of l-cysteine from 3-phosphoglycerate (fig. 1), and our analyses suggested that it was acquired through LGT from bacteria. This protein was already present in the last Fornicata common ancestor but lost in the last *Diplomonadida* common ancestor (supplementary fig. 24, Supplementary Material online). Synthesis from l-glycine shows a more complex history. Our analyses suggested that serine hydroxymethyltransferase was ancestral to Fornicata (supplementary fig. 25, Supplementary Material online). However, diplomonad sequences grouped with bacteria in our phylogenetic analyses, which suggests that this protein was lost in the last *Diplomonadida* common ancestor and then reacquired from bacteria in the lineage leading to *S. salmonicida* and *Trepomonas* sp. PC1 (supplementary fig. 25, Supplementary Material online). The phylogenetic tree of serine *O*-acetyltransferase showed an acquired origin from cyanobacteria (supplementary fig. 26, Supplementary Material online). Interestingly, cysteine synthase, which is the only protein common in both pathways, was acquired via two independent LGT events from bacteria in the diplomonad and *K. bialata* lineages (supplementary table 1 and supplementary figs. 27 and 28, Supplementary Material online). The diplomonad cysteine synthase gene was acquired before the split between *D. brevis* and diplomonads and later lost in the lineages leading to *Giardia*.

Glutathione reduces ^•^OH and lipid radicals or acts as a cofactor in glutaredoxin (fig. 2*a* and *b*). It is synthesized by glutamate-cysteine synthase (GSH1) and glutathione synthetase (GSH2) (fig. 2*b*). Although it is thought that cysteine is the major nonprotein thiol in microaerophilic protists (Krauth-Siegel and Leroux 2012), functional genes for GSH1 and GSH2, as well as a glutaredoxin-like protein, have been detected in *Giardia* species (Morrison et al. 2007). Homologs of these genes were found in *K. bialata* but not in *S. salmonicida* (fig. 1). In most species, glutathione is reduced by the enzyme glutathione reductase, but no glutathione reductase homologs were found in any Fornicata species. The protein thioredoxin reductase can reduce glutathione in *G. intestinalis* (Brown et al. 1996b), and we propose the same mechanism for reducing glutathione in the other Fornicata species (fig. 2*b*).

Our phylogenetic analyses were inconclusive about the origin of this pathway. Fornicata GSH1 clustered as a sister group to *Blastocystis* with strong support, distant from other Excavata and SAR homologs (supplementary fig. 29*a* and *b*, Supplementary Material online), which suggested that GSH1 has undergone at least one eukaryote-to-eukaryote LGT event. However, we cannot establish the direction of this event. We assume that a homolog of GSH2 was present in the last Fornicata common ancestor, even though our analysis was inconclusive (fig. 4 and supplementary fig. 30*a* and *b*, Supplementary Material online). GSH1 produces l-γ-glutamylcysteine, the only known cellular role of which is as a precursor of glutathione. The only known exception is that Halobacteria use l-γ-glutamylcysteine as an antioxidant through the enzyme bis-γ-glutamylcysteine reductase (Kim and Copley 2013). We did not detect homologs of this enzyme in any of the fornicata genomes. Our data supported that GSH1 synthesized l-γ-glutamylcysteine in the last Fornicata common ancestor and that this molecule was the substrate for a GSH2 (fig. 4).

The glutaredoxin-like protein plays different roles within the cell. This protein is involved in the FeS assembly and also rearranges nonnative disulfide bonds in proteins (Rada et al. 2009; Krauth-Siegel and Leroux 2012). Unfortunately, the glutaredoxin-like data matrix did not contain enough signal to reveal the evolutionary history of the protein. PDI, thioredoxin reductase, and thioredoxin are additional proteins that could be involved in the reduction of oxidized cysteine residues (fig. 2*a*).

### Glutathione Peroxidase: H_2_O_2_ Detoxification and Restoration of Lipids

Glutathione peroxidase uses two molecules of glutathione to reduce H_2_O_2_ to H_2_O or lipid hydroperoxides to their stable lipid alcohol state (Mannervik 1985) (fig. 2*a*). Homologs of this protein were found in *K. bialata*, *D. brevis*, and *Ergobibamuscyprinoides*. The final data matrix was composed mainly of bacterial sequences (supplementary fig. 31, Supplementary Material online), and there were only six other eukaryotic sequences in the final data matrix. These other six sequences clustered together separate from Fornicata with strong support. Our analyses suggested the acquisition of a glutathione peroxidase from bacteria early in the evolution of Fornicata and its subsequent loss in the last *Diplomonadida* common ancestor (fig. 4).

## Discussion

We have combined available biochemical data with genomic and phylogenetic methods in an approach similar to that which has been previously used to study the evolution of the mitochondrion-related organelles (Nývltová et al. 2015; Degli Esposti et al. 2016; Leger et al. 2017). We complemented that approach with gene expression data from cells under oxygen stress conditions (Ma’ayeh et al. 2015). We identified a set of proteins involved in detoxification of ROS (fig. 1) and proposed a putative oxygen defense pathway in Fornicata (fig. 2*a* and *b*). Several of these enzymes have high specificity for their respective ROS (Di Matteo et al. 2008; Mastronicola et al. 2010, 2014; Testa, Mastronicola, et al. 2011; Castillo-Villanueva et al. 2016). For a eukaryote that is adapted to low-oxygen levels, having a gene coding for an oxygen-scavenging enzyme that works at low substrate concentrations probably has a selective advantage.

### Evolution of the Oxygen Detoxification Pathway

Based on the phylogenetic analyses of these proteins, we propose a stepwise evolutionary transition of the oxygen detoxification pathway characterized by constant evolutionary change from the last Fornicata common ancestor to the extant species included in this study, and where proteins have been gained and lost (fig. 4). The evolutionary history of rubrerythrin is an example of this dynamic situation. This enzyme was acquired before the divergence of *K. bialata*, most likely lost in the last *Diplomonadida* common ancestor, and reacquired in the lineage leading to *S. salmonicida* and *Trepomonas*. Interestingly, anaerobic ribonucleotide reductase has previously been reported to have a similar evolutionary history with repeated losses and acquisitions in diplomonads (Xu et al. 2016).

Serine hydroxymethyltransferase, one of the two proteins that seems to be ancestrally present in Fornicata, is another example of the dynamic process of gene loss and gain. This protein is potentially localized in the hydrogenosomes of the analyzed species (Schneider et al. 2011; Jerlström-Hultqvist et al. 2013; Leger et al. 2017; Tanifuji et al. 2018). Our analyses supported that serine hydroxymethyltransferase was not present in the last *Diplomonadida* common ancestor, but its function was reacquired in the hydrogenosomes of the ancestor of *S. salmonicida* and *Trepomonas* sp. PC1 via LGT. Acquiring a missing hydrogenosome function via LGT has been previously demonstrated by Leger et al. (2017), who showed that *S. salmonicida* has laterally acquired the hydrogenosomal acetyl-CoA synthase. Our analysis also provided an evolutionary explanation for the observation that *G. intestinalis* lacks SOD (Brown et al. 1995), which is that its acquisition of SOR (a prokaryotic enzyme that performs the same function as SOD) most likely led to the loss of SOD in the ancestor of *D. brevis* and diplomonads due to functional redundancy.

LGT can be a powerful evolutionary mechanism for metabolic adaptation of key processes in eukaryotic cells (Alsmark et al. 2013; Karnkowska et al. 2016; Xu et al. 2016; Eme et al. 2017; Stairs et al. 2018). However, it can be difficult to detect LGT through single-gene phylogenetic analyses. Assembling a suitable data matrix is not trivial and weakly supported resolution in the resulting phylogenetic trees can make it difficult to distinguish between different interpretations of the evolutionary history of a protein family. In our case, four proteins (small proteins with little sequence conservation, Pan and Bardwell 2006) failed to produce reliable alignment. Our analyses also failed to identify the evolutionary history of both proteins involved in the synthesis of glutathione because the evolutionary signal was weak, and the phylogenetic support did not change after the long-branch artifact detection tests (supplementary figs. 29 and 30, Supplementary Material online). In all these cases, the proteins were classified to have an unclear origin. It can also be difficult to specify the donor species in an LGT event. In five cases, we identified the putative bacterial donor group, but in all other cases, the bacteria were mixed indicating multiple LGT events among them. None of the genes showed affinity to *Alphaproteobacteria* which is expected if they arrive to the nucleus via mitochondrial gene transfer (Timmis et al. 2004).

### Lateral Gene Transfers between Eukaryotic Lineages

The mechanism (or mechanisms) of how LGT between eukaryotes happens is not well established, but most of the reported cases occurred between lineages that occupy the same ecological niche (Andersson et al. 2003; Alsmark et al. 2013; Grant and Katz 2014; Eme et al. 2017). In our study, we identified three cases of gene transfers between parasitic lineages adapted to intestinal tract of animals. First, *G. intestinalis* flavohemoprotein homologs form a sister group to four members of Trypanosomatidae (supplementary fig. 18, Supplementary Material online), which are gut parasite of insects (Runckel et al. 2014; Kraeva et al. 2015; Flegontov et al. 2016) and related to *Leishmania*, a vertebrate parasite that uses insects as a vector (Ivens et al. 2005). Second, the Fornicata GSH1 homologs are sister group of one *Blastocystis* sequence (supplementary fig. 29, Supplementary Material online). This relation between the *Blastocystis*, a common inhabitant of the intestinal tract of animals, and Fornicata (specially *G. intestinalis* and *S. salmonicida*) has been described before (Eme et al. 2017). Third, nitroreductase has been transferred from diplomonads to *Entamoeba* (supplementary fig. 3, Supplementary Material online). Cases of LGT between these two groups have also been described before (Andersson et al. 2003; Hampl et al. 2008; Grant and Katz 2014).

### Oxygen Detoxification Pathway in the Last *Diplomonadida* Common Ancestor and the Last Fornicata Common Ancestor

Most of the proteins with an unclear origin were present in the last Fornicata common ancestor. This highlighted how difficult it is to detect ancient LGT events. Nevertheless, more recently acquired genes were resolved with stronger support, making it possible to understand the assembly and modification of the oxygen detoxification pathway over evolutionary timescales within Fornicata. We were able to reconstruct a putative pathway in the last *Diplomonadida* common ancestor (fig. 4). It is likely that this ancestor was already adapted to low-oxygen levels, already had a complete oxygen detoxification pathway with most of the enzymes that we observed in the extant diplomonads, and was able to synthesize both glutathione and l-cysteine. We observed a clear difference in nonprotein thiols usage in extant diplomonads. Our analyses suggested the loss of all components for the synthesis of l-cysteine in the ancestor of Giardiinae, whereas the ancestor of Hexamitinae lost the glutaredoxin-like protein and the capacity to synthesize glutathione. These differential losses might be due to the different environments that these species inhabit. Giardiinae species are gut parasites and they can take in amino acids directly from the host’s diet, whereas Hexamitinae species have more diverse lifestyles, from free living to parasites of different tissues of the host. Even when they take l-cysteine from the diet, having a pathway to synthesize l-cysteine can be advantageous for them. Surprisingly, a mechanism for restoring oxidized methionine residues was inferred to be lacking in the last *Diplomonadida* common ancestor.

The oxygen detoxification pathways of the closely related intestinal parasites *G. intestinalis* and *G. muris* are very similar. Only one additional protein, flavohemoprotein, is involved in the *G. intestinalis* oxygen detoxification pathway (fig. 4). The acquisition of this protein might have been advantageous for this species. NO is produced by intestinal epithelial cells, macrophages, neutrophils, and natural killer cells as a defense against infections and as a signal of the inflammatory process (Tripathi et al. 2007). Being able to remove NO efficiently from the environment would be important for a parasite, allowing it to avoid damage by NO and O_2_ and to reduce the immune response of the host. A-type flavoprotein can usually detoxify both O_2_ and NO (Saraiva et al. 2004), although in *G. intestinalis* this enzyme has a preference for O_2_ (Di Matteo et al. 2008), whereas flavohemoprotein detoxifies NO (Mastronicola et al. 2010). It could be that A-type flavoprotein homologs are able to detoxify both toxic elements in other Fornicata, but this dual function was lost in *G. intestinalis* homologs with the acquisition of flavohemoprotein. If this hypothesis is correct, it could explain how other species of Fornicata are able to detoxify NO without a flavohemoprotein.

The oxygen detoxification pathway in the free-living *K. bialata* is similar to the pathway in the diplomonad ancestor (fig. 4) but with more functional redundancy. Our analyses showed that at least seven protein families were acquired independently in the *K. bialata* lineage, which led to the convergence of oxygen detoxification pathways in which proteins of different origins performed analogous functions in different Fornicata lineages. Such examples of convergence have indeed been observed before within microbial eukaryotes. For example, kinetoplastids have independently acquired a protein for the biosynthesis of heme groups in three different lineages (Cenci et al. 2016). We observed more versatility in *K. bialata* with the use and synthesis of nonprotein thiols, such as the ability to synthesize both l-cysteine and glutathione. *Kipferliabialata* also has a glutaredoxin-like protein and a glutathione peroxidase, both of which use glutathione as a cofactor. This diversity in the use of nonprotein thiols is likely to be advantageous for a free-living lifestyle. Although parasitic species can take up amino acids and small peptides from a host, free-living species must depend on the bacteria they eat and the compounds they can synthesize.

Our analyses revealed, at least in part, the processes of oxygen detoxification in the last Fornicata common ancestor (fig. 4). We identified four proteins (A-type flavoprotein, phosphoserine aminotransferase, FAD/FMN-dependent oxidoreductase, and SOD) laterally acquired before the diversification of the group (fig. 4). Our analyses suggest that this ancestor could not synthesize l-cysteine, but used glutathione and reduced O_2_ to H_2_O using a pathway similar to that currently used by diplomonads and *K. bialata*. The oxygen detoxification pathway could have used fewer proteins than are encoded in extant Fornicata genomes, although it is of course possible that additional proteins present in the Fornicata ancestor were lost in all currently sampled lineages. Additional genome sequences from other members of Fornicata could help fill the gaps of the ancestral oxygen detoxification pathway (e.g., glutathione, MsrA, and MsrB recycling) and help elucidate the evolutionary history of the many other proteins whose histories remain unclear. We hope that some of the hypothesis proposed here will be tested experimentally and by doing so generate new biochemical knowledge on these processes and might reveal additional proteins that detoxify O_2_ and ROS.

## Supplementary Material

Supplementary data are available at *Genome Biology and Evolution* online.

## Supplementary Material

evz188_Supplementary_Data

## Notes

**Data deposition:** This project has been deposited at GenBank under the accessions MG708240-MG208272.
