# Supplementary material for: Lateral Acquisitions Repeatedly Remodel the Oxygen Detoxification Pathway in Diplomonads and Relatives
Source: Genome Biol Evol. 2019 Sep 5;11(9):2542–56. doi: 10.1093/gbe/evz188 (PMC6934886; doi:10.1093/gbe/evz188)
Supplement: evz188_Supplementary_Data [file evz188_supplementary_data.zip › Supplementary_table_1_&_supplementary_figures_1_31.pdf]

Lateral acquisitions repeatedly remodel the oxygen detoxification pathway in diplomonads and relatives

Alejandro Jiménez-González[1], Feifei Xu[2], Jan O. Andersson[1, *]

[1]Uppsala Biomedicine Centre, Department of Cell and Molecular Biology, Molecular Evolution program, Uppsala University, Husargatan 3, Uppsala, Box 596, 751 24, Sweden

[2]Uppsala Biomedicine Centre, Department of Cell and Molecular Biology, Microbiology program, Uppsala University, Husargatan 3, Uppsala, Box 596, 751 24, Sweden

[*]Author for Correspondence: Jan O. Andersson, Department of Cell and Molecular Biology, Molecular Evolution program, Uppsala University, Uppsala Sweden, jan.andersson@icm.uu.se

Supplementary Table 1: Complete list of pairwise calculations of the fraction of BLASTP hits in common for every protein family (%). The fraction was calculated using Cd-hit-2d with the default setup.

	Pairs	Fraction of Blastp hits in common (%)
**A-type flavoprotein**	Diplomonads-*Kipferlia*	75
**Cysteine synthase**	Diplomonads-*Kipferlia*	17
**FAD/FMN dependent oxidoreductase**	*Giardia-Spironucleus1*	20
	Giardia-Spironucleus2	1
	Giardia-Kipferlia1	16
	Giardia-Kiperlia2	0
	Kipferlia1-Spironucleus1	33
	Kipferlia1-Spironucleus2	2
	Kipferlia1-Kipferlia2	0
	Kipferlia2-Spironucleus1	0
	Kipferlia2-Spironucleus2	59
**FixW**	Diplomonads-*Kipferlia*	24
**Flavohemoprotein**	Only present in *G. intestinalis*	-
**Glutaredoxin**	Diplomonads-*Kipferlia*	52
**Glutathione peroxidase**	Only present in *K. bialata*	-
**GSH1**	Diplomonads-*Kipferlia*	67
**GSH2**	Diplomonads-*Kipferlia*	22
**Hybrid cluster protein**	Diplomonads-*Kipferlia*	99
**MsrA**	Diplomonads-*Kipferlia*	0
**MsrB**	*Giardia-Spironucleus*	0
	Giardia-Kipferlia	4
	Kipferlia-Spironucleus	0
**NADH oxidase**	Diplomonads-*Kipferlia*	0
**NADPH oxidoreductase**	*Giardia-Spironucleus*	12
	Giardia-Kipferlia	19
	Kipferlia-Spironucleus	15
**Nitroreductase**	Diplomonads-*Kipferlia*	68
**PDI**	Diplomonads-*Kipferlia*	23
**Peroxiredoxin**	Diplomonads-*Kipferlia*	76
**Phosphoserine aminotransferase**	Only present in *Kipferlia*	-
**Rubrerythrin**	Diplomonads-*Kipferlia*	85
**Serine hydroxymethyltransferase**	Diplomonads-*Kipferlia*	37
**Serine O-acetyltransferase**	Diplomonads-*Kipferlia*	61
**SOD**	Only present in *Kipferlia*	-
**SOR**	Only present in diplomonads	-
**Thioredoxin**	Only present in *Kipferlia*	-
**Thioredoxin reductase**	Diplomonads-*Kipferlia*	33

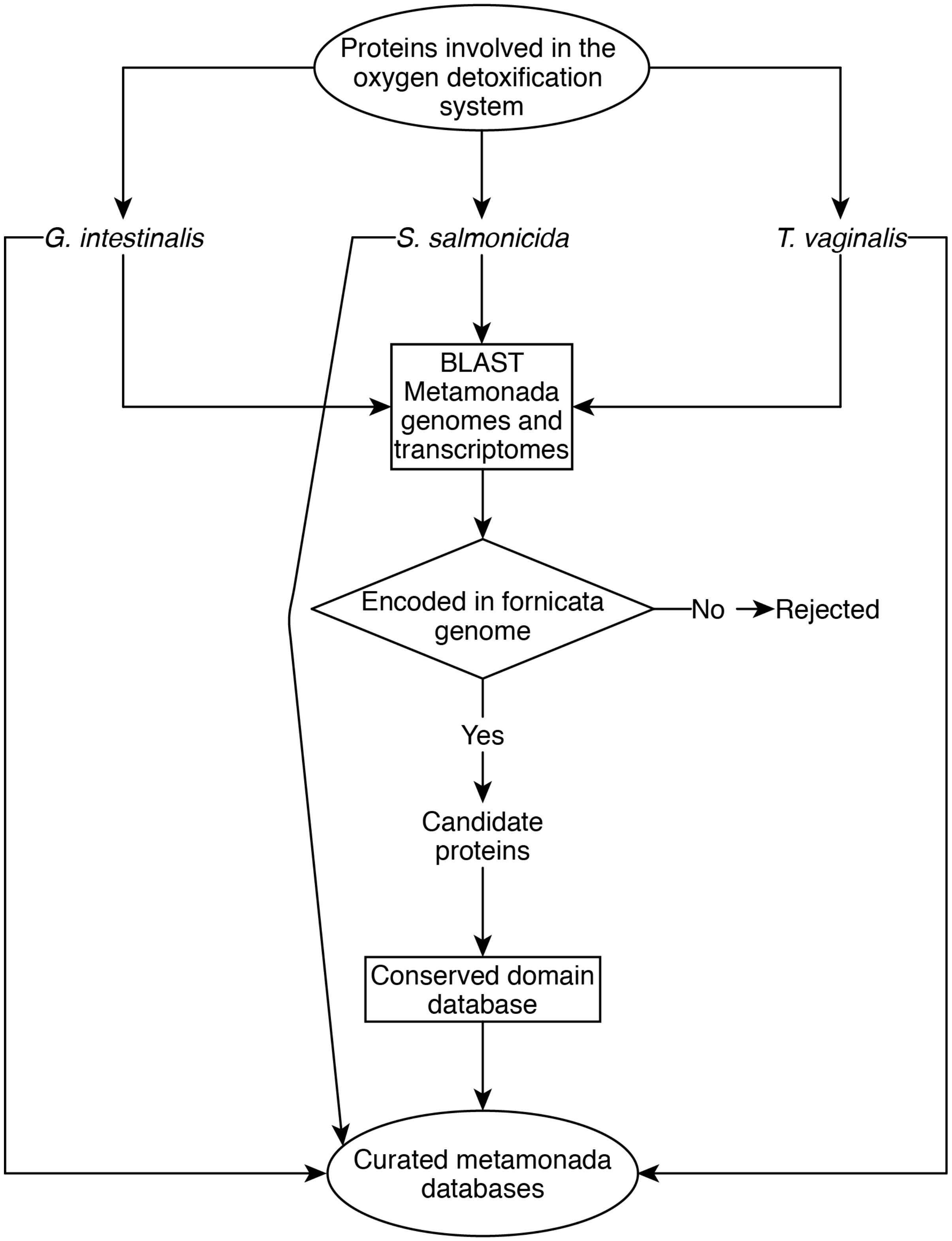

Supplementary Figure 1: Workflow of the identification of proteins involved in the oxygen detoxification system in Fornicata and creation of curated metamonada databases. Only proteins encoded in at least one fornicata genome were included in the oxygen detoxification pathway.

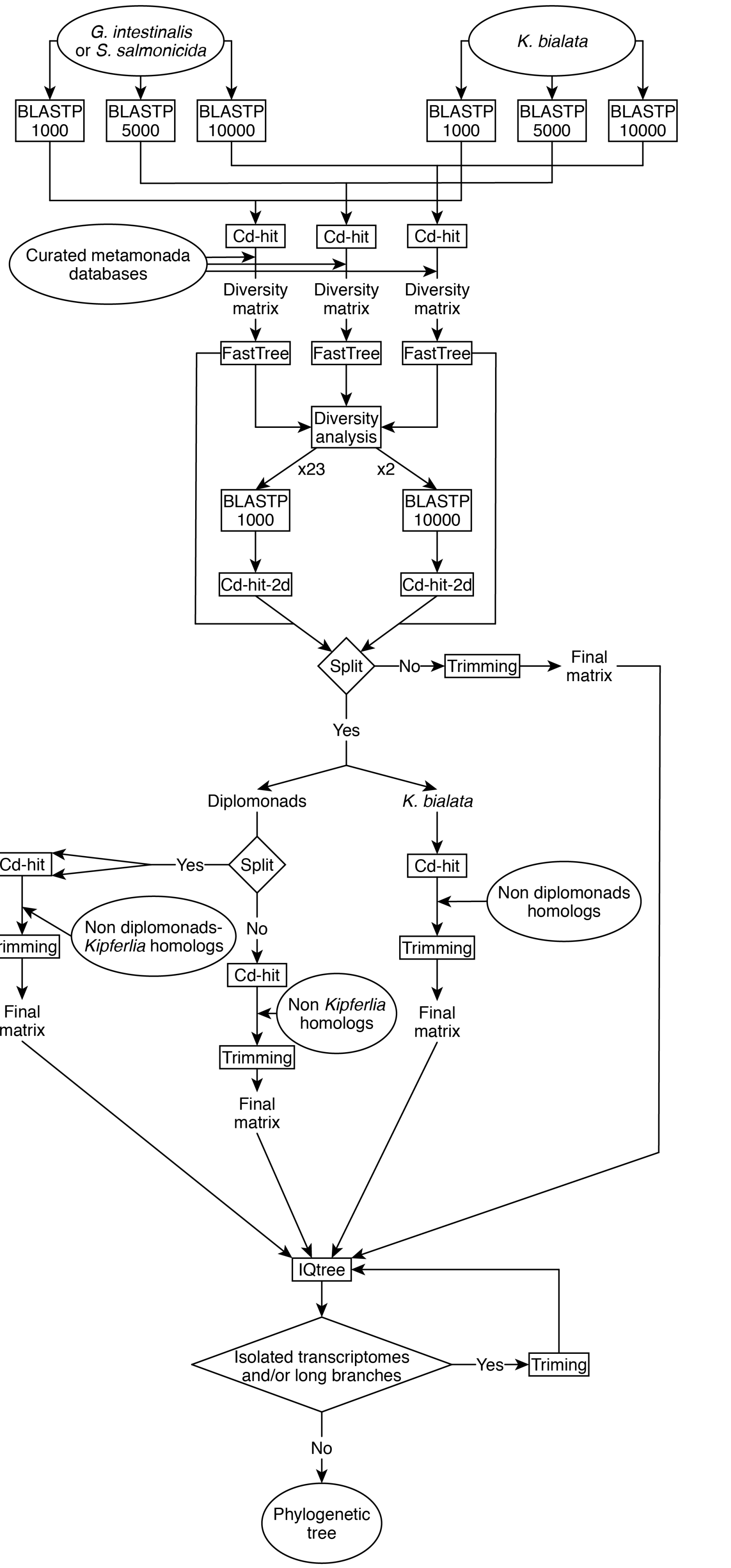

Supplementary Figure 2: Workflow for the assembly of datasets, trimming and phylogenetic analyses.

Supplementary figure 3: Nitroreductase

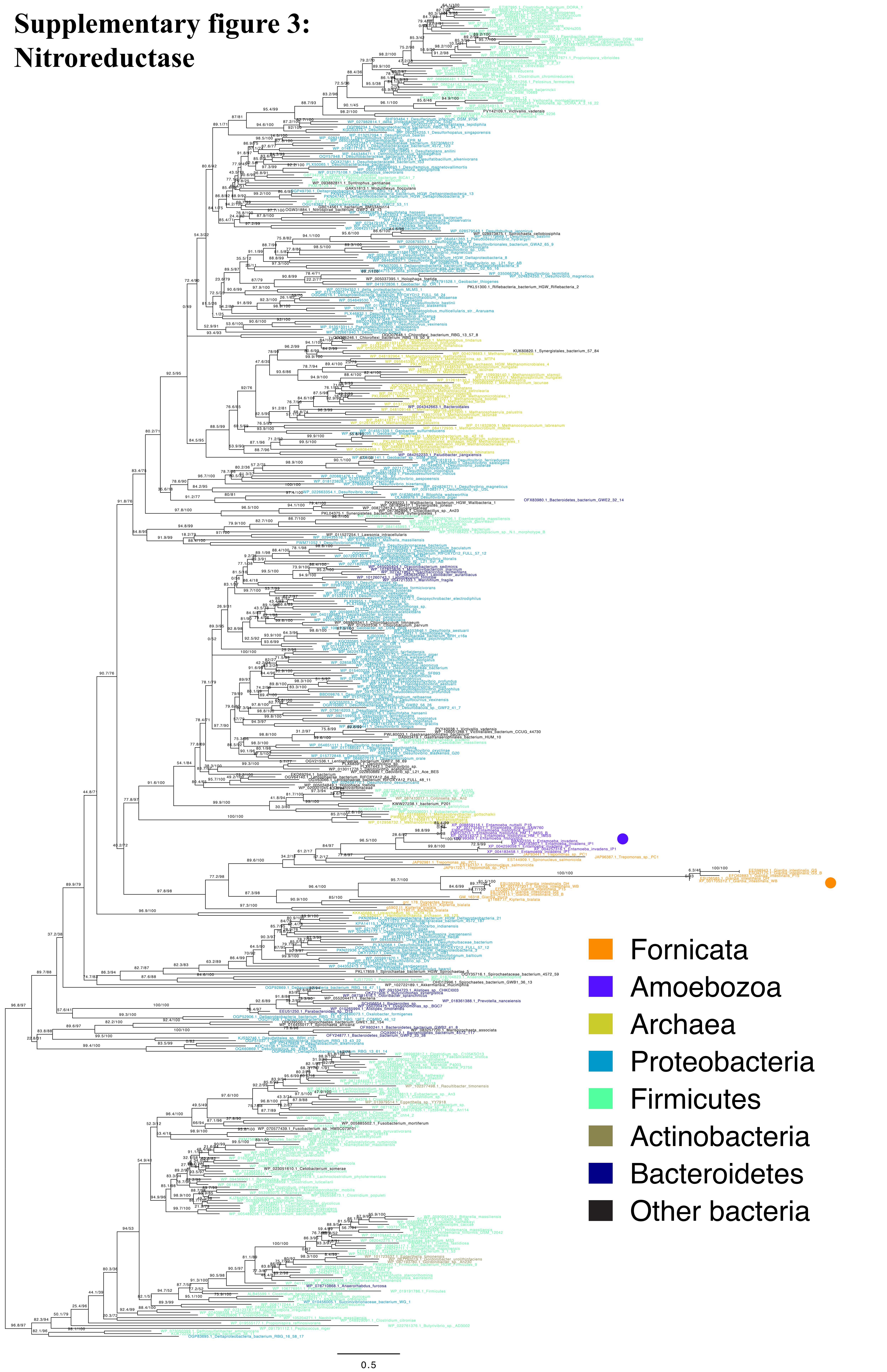

Supplementary figure 4a: FAD/FMN dependent oxidoreductase 1

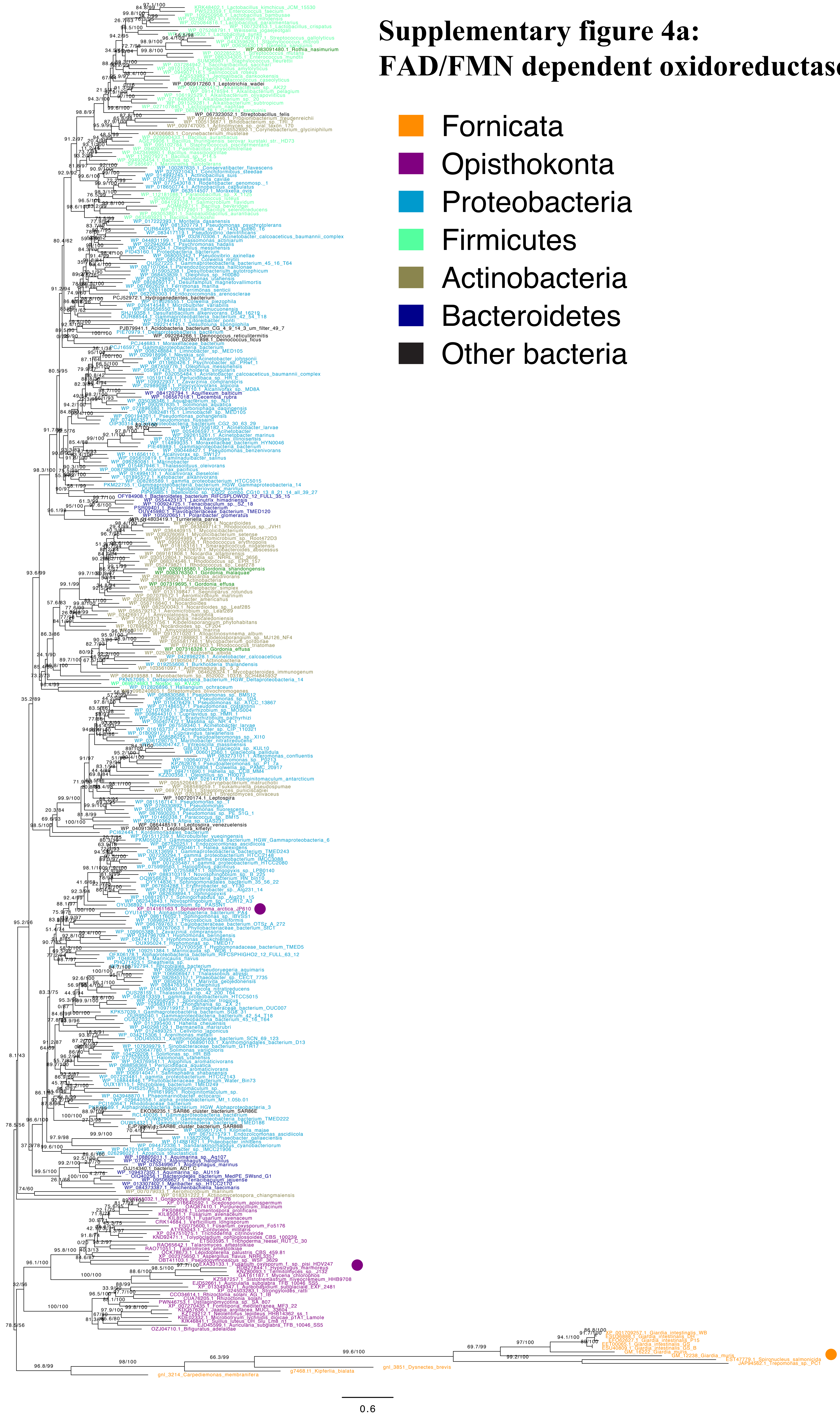

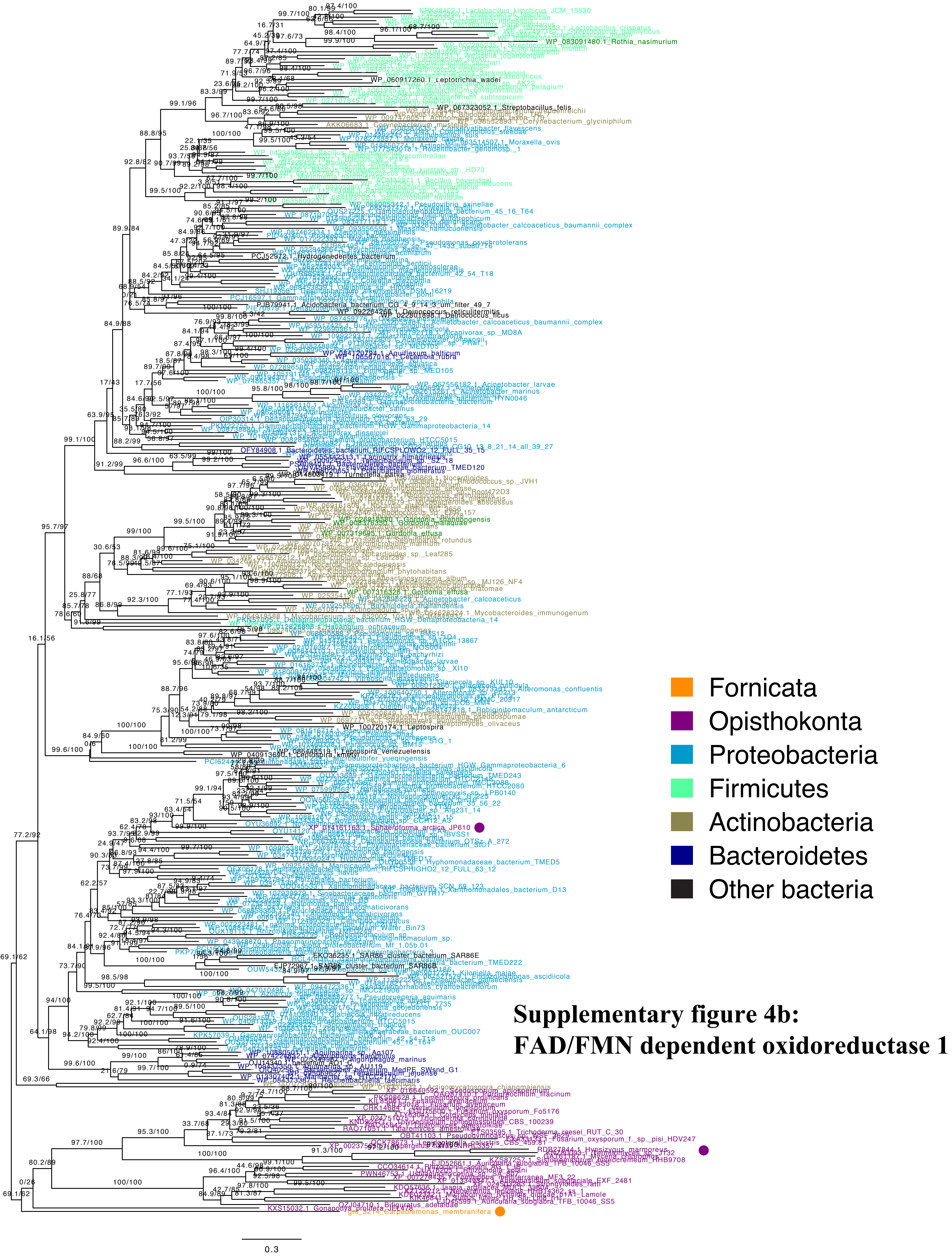

Supplementary figure 4b: FAD/FMN dependent oxidoreductase 1

Supplementary figure 5: FAD/FMN dependent oxidoreductase 2

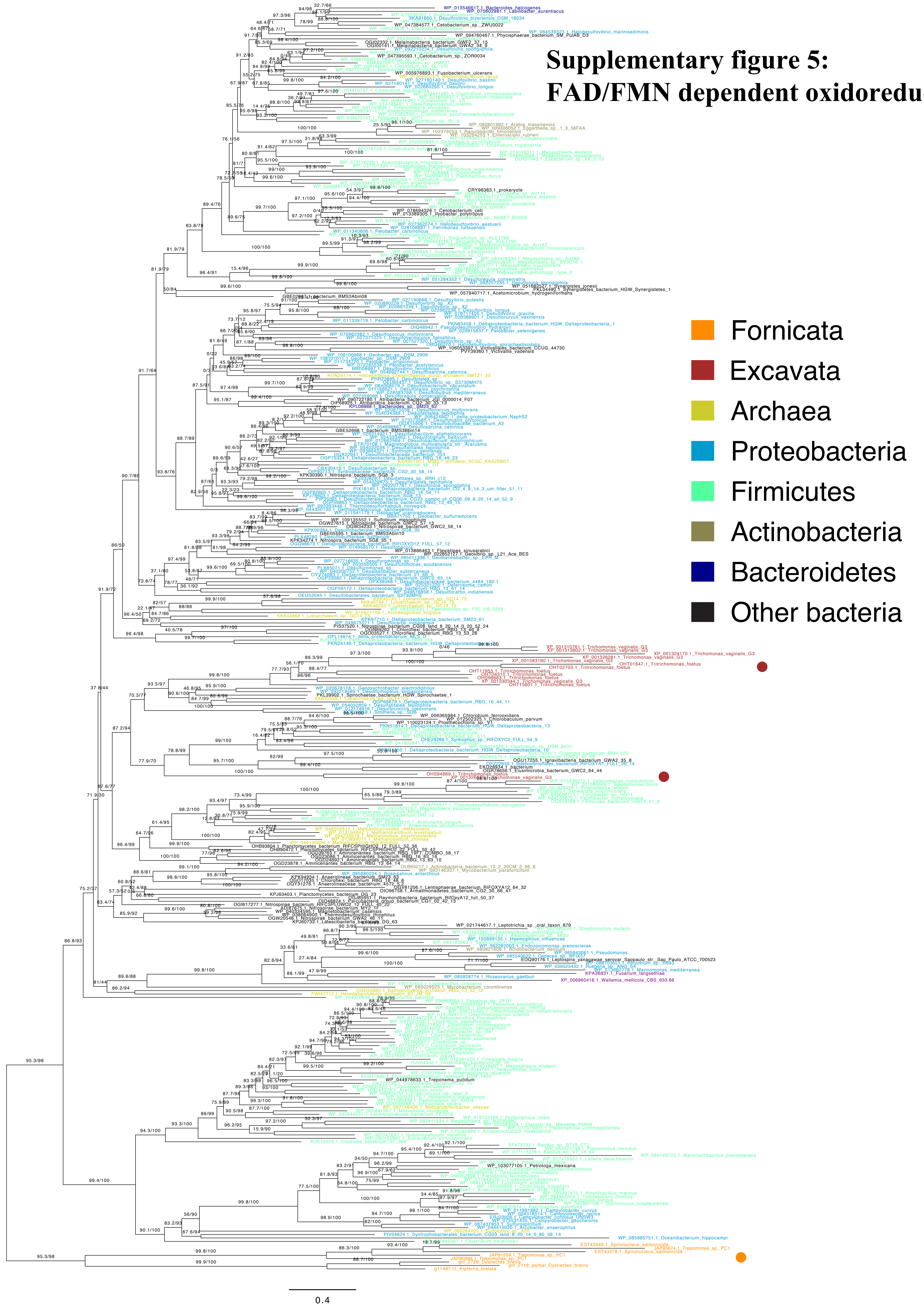

Supplementary figure 6: NADPH oxidoreductase *Giardia*

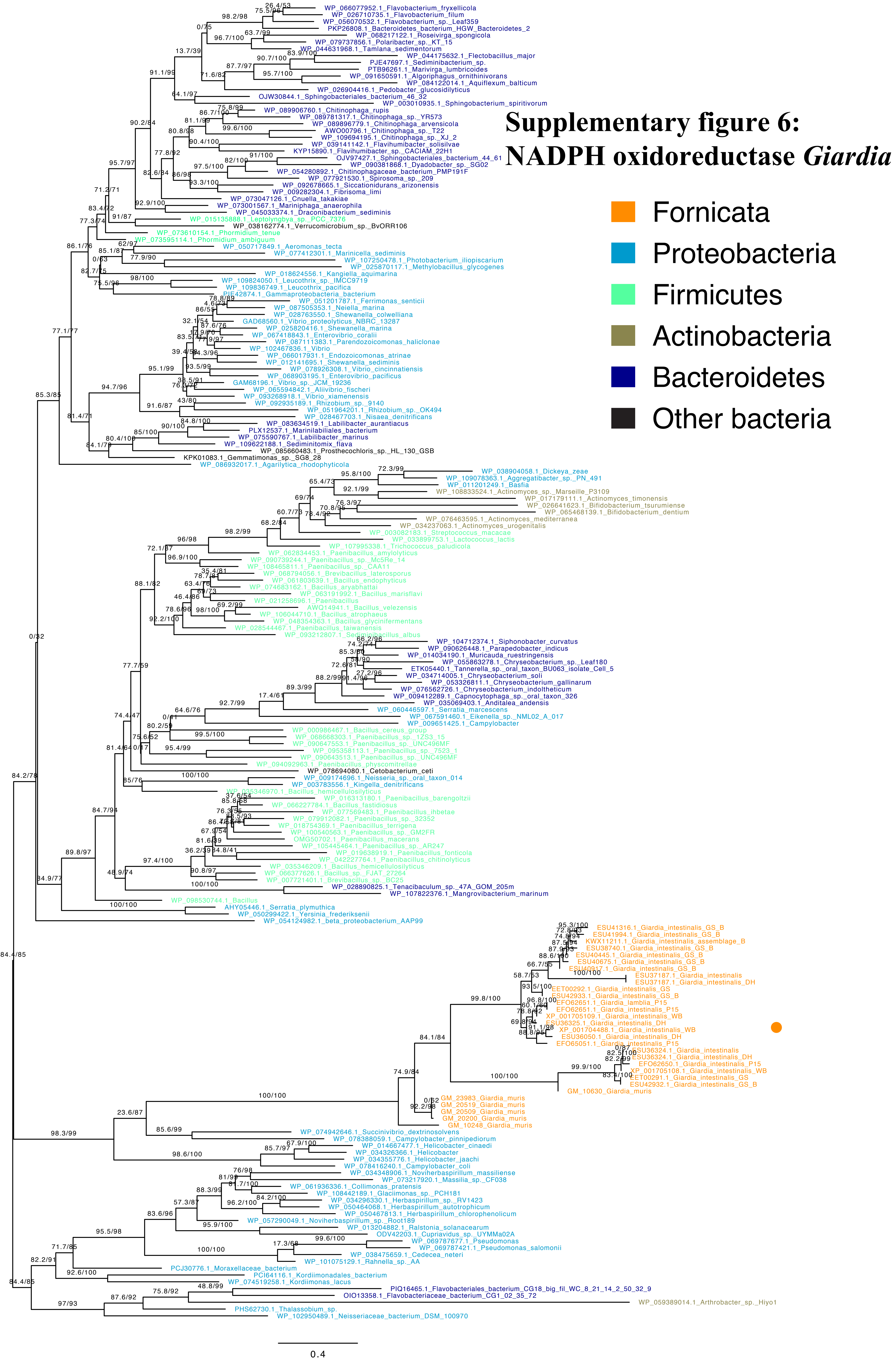

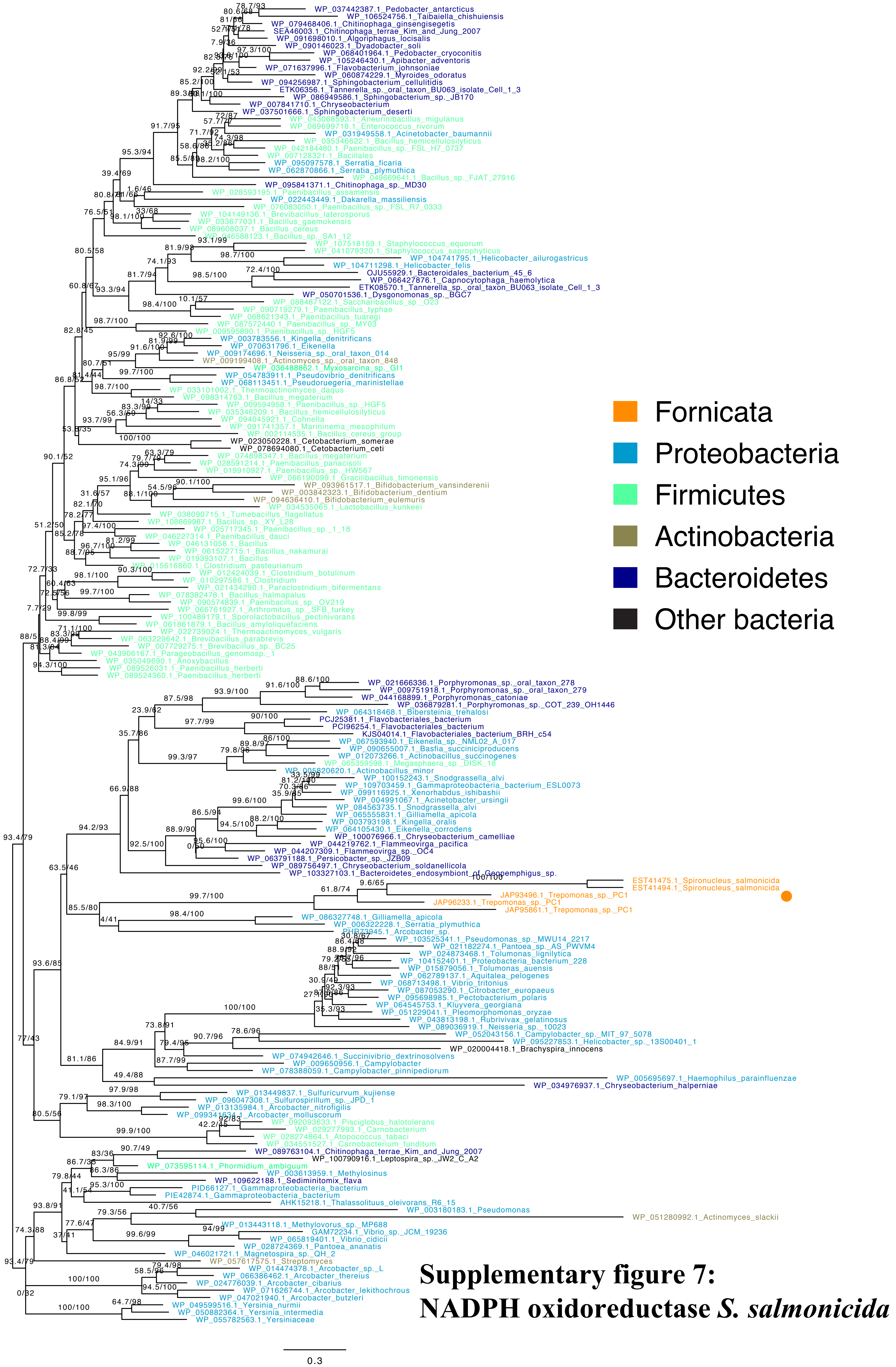

Supplementary figure 7: NADPH oxidoreductase *S. salmonicida*

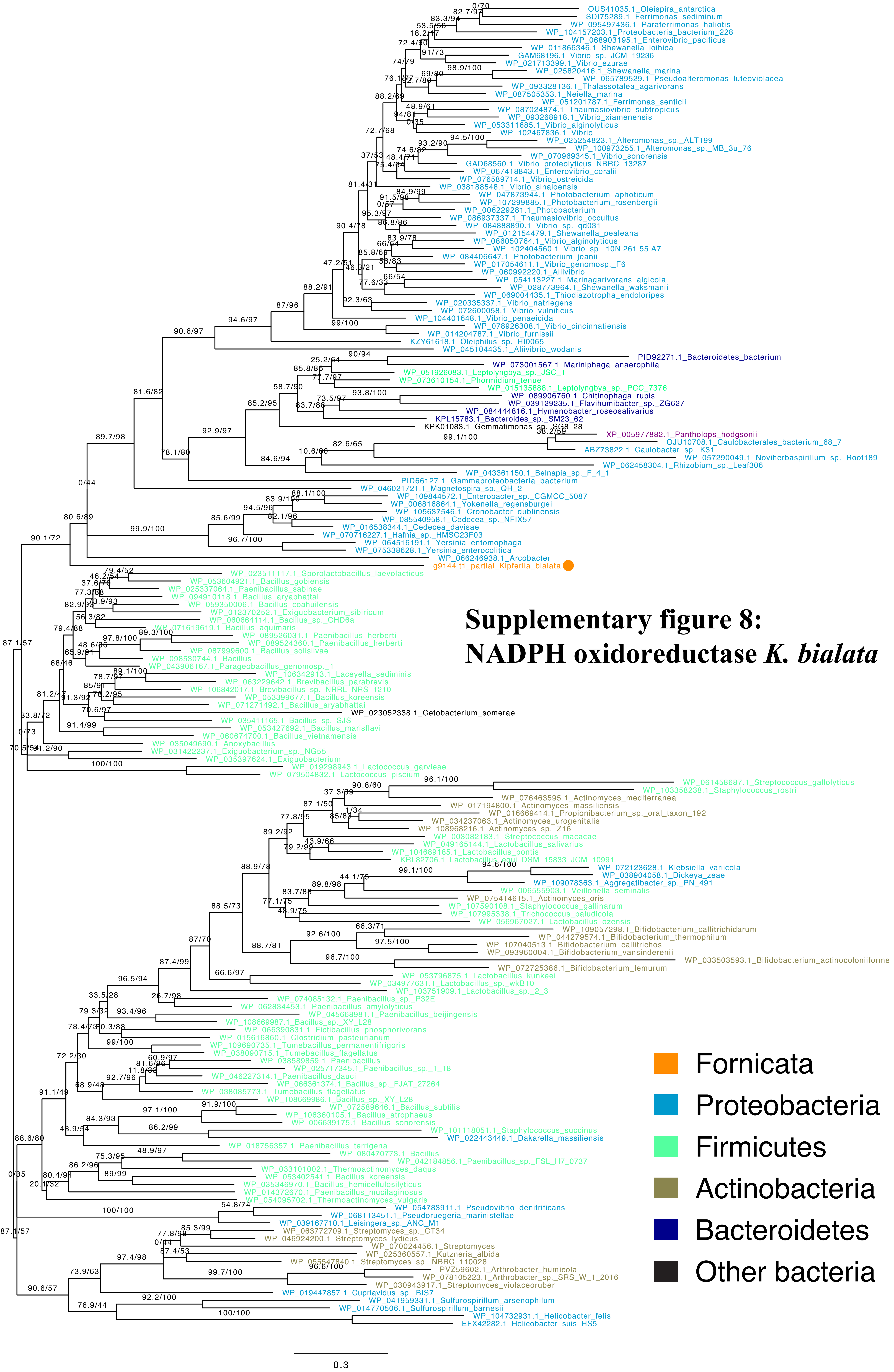

Supplementary figure 8: NADPH oxidoreductase *K. bialata*

Supplementary figure 9: Superoxide dismutase

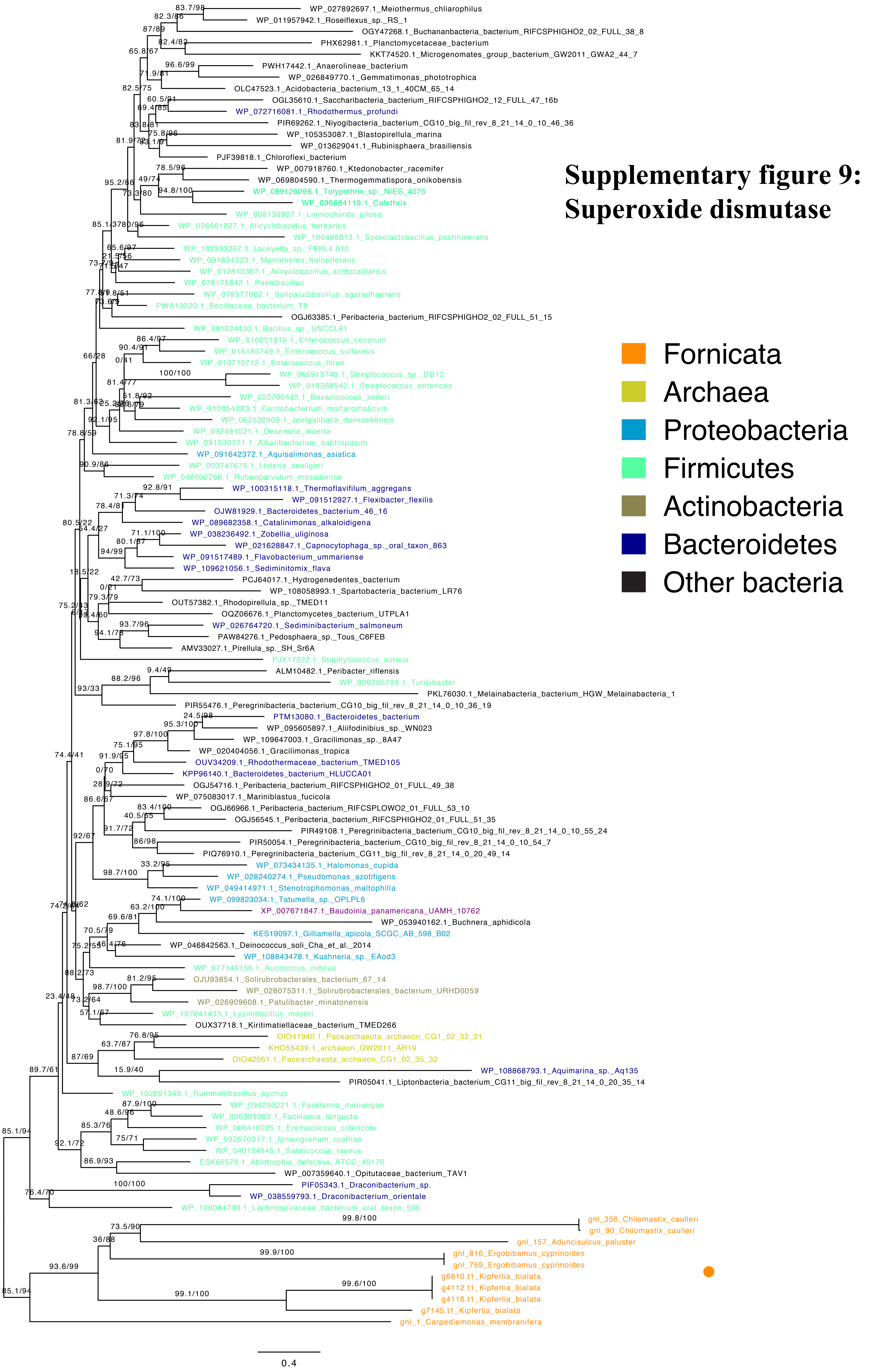

Supplementary figure 10: Superoxide reductase

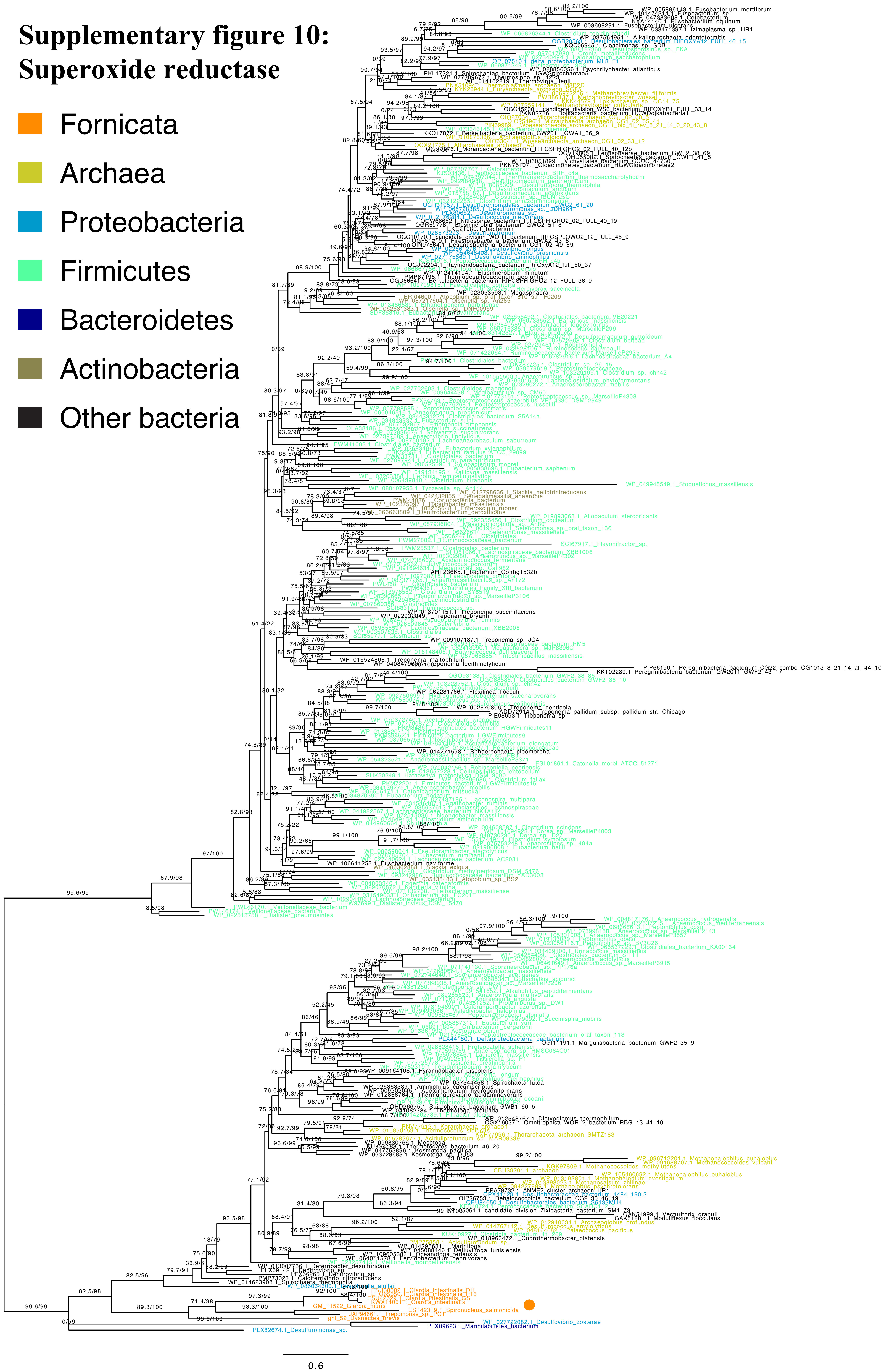

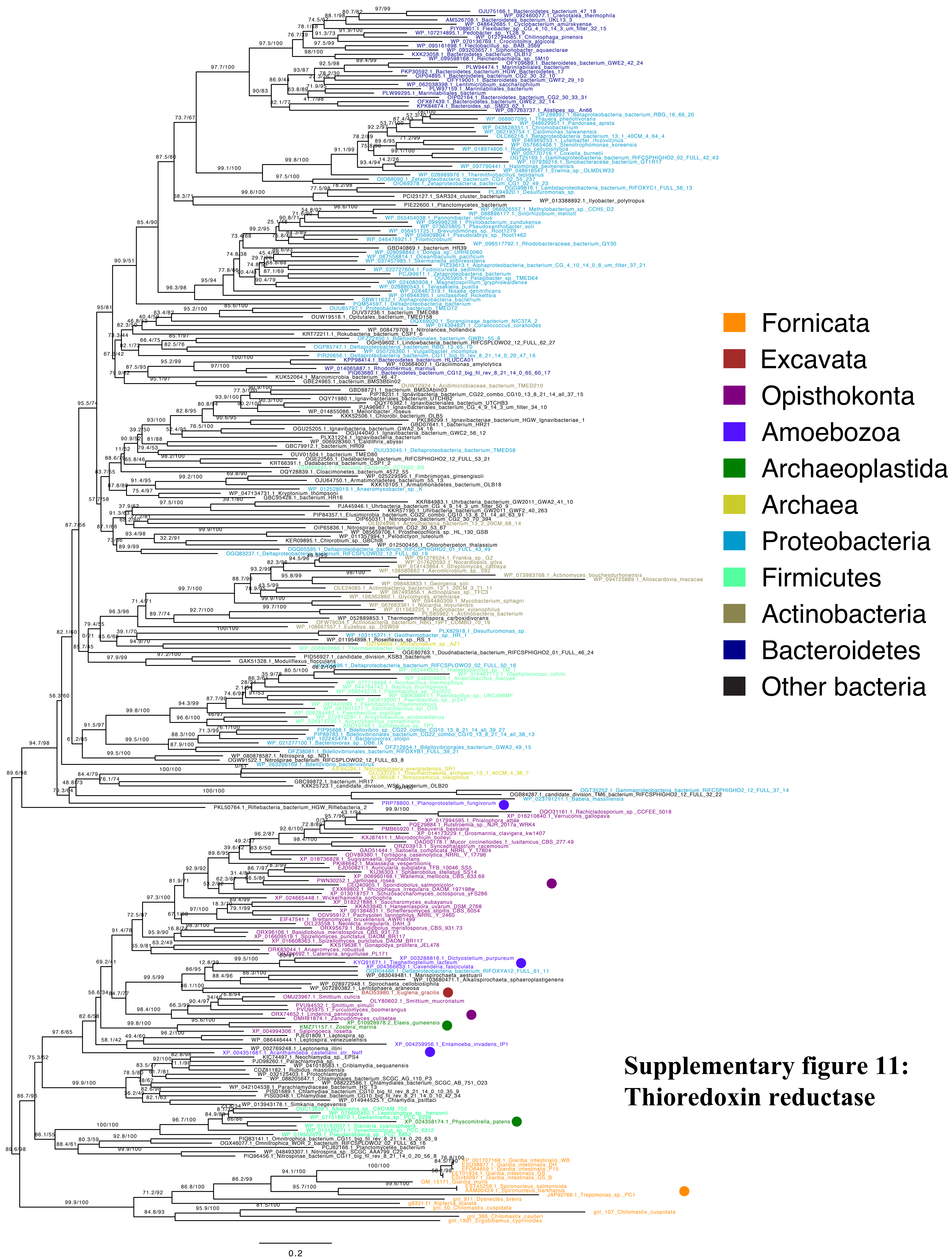

Supplementary figure 11: Thioredoxin reductase

Supplementary figure 12a: Peroxiredoxin

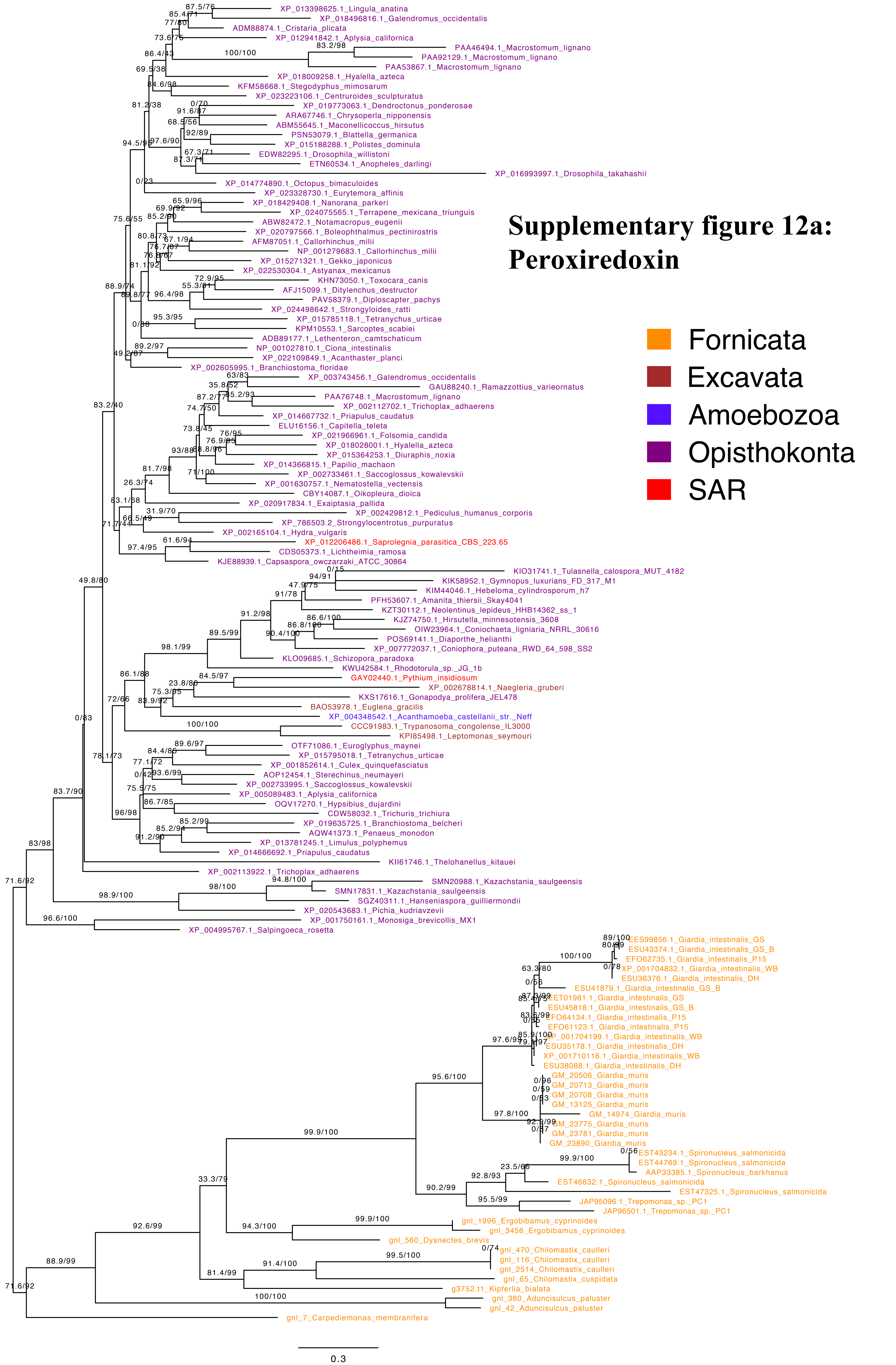

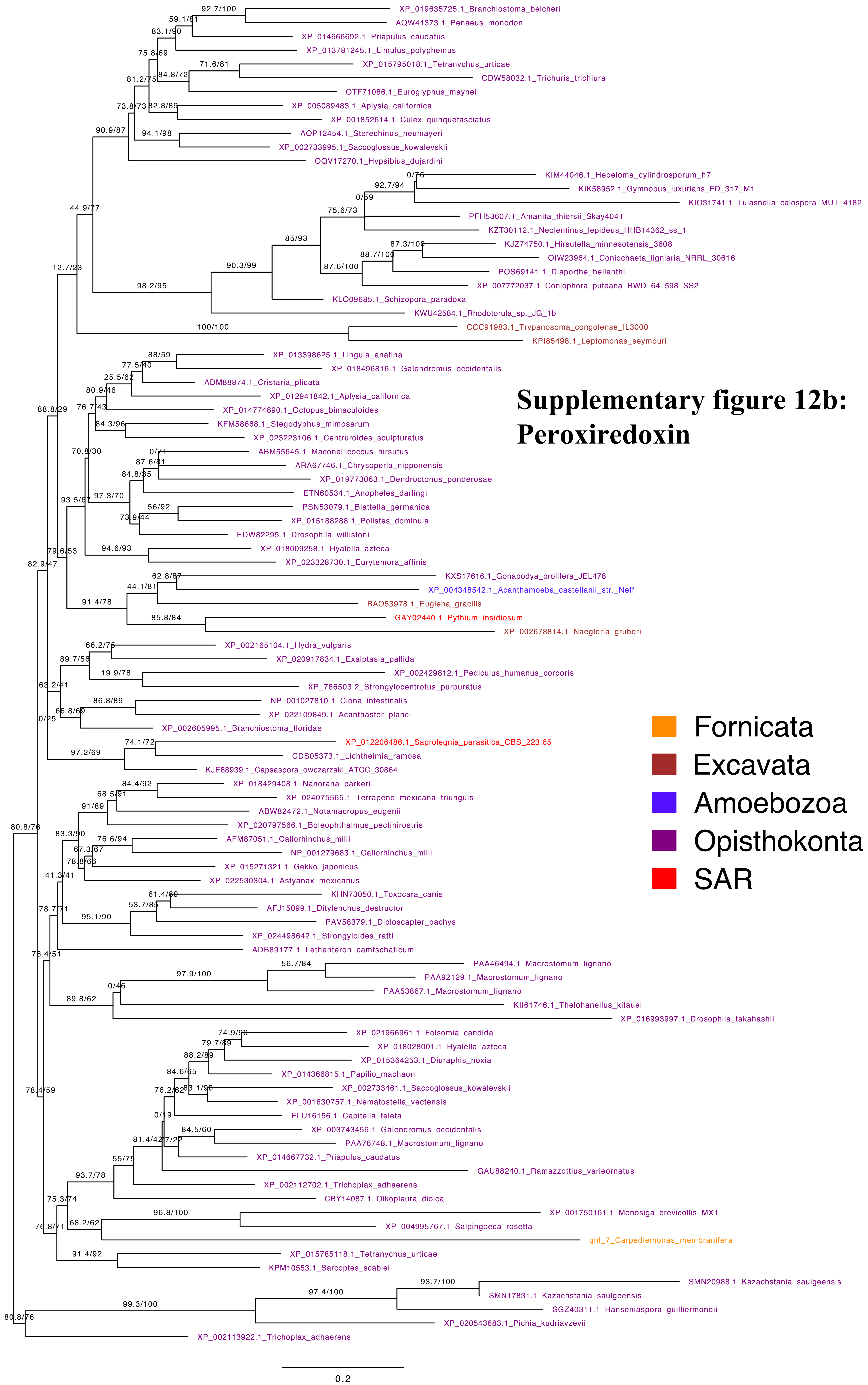

Supplementary figure 12b: Peroxiredoxin

Supplementary figure 13: Hybrid-cluster protein

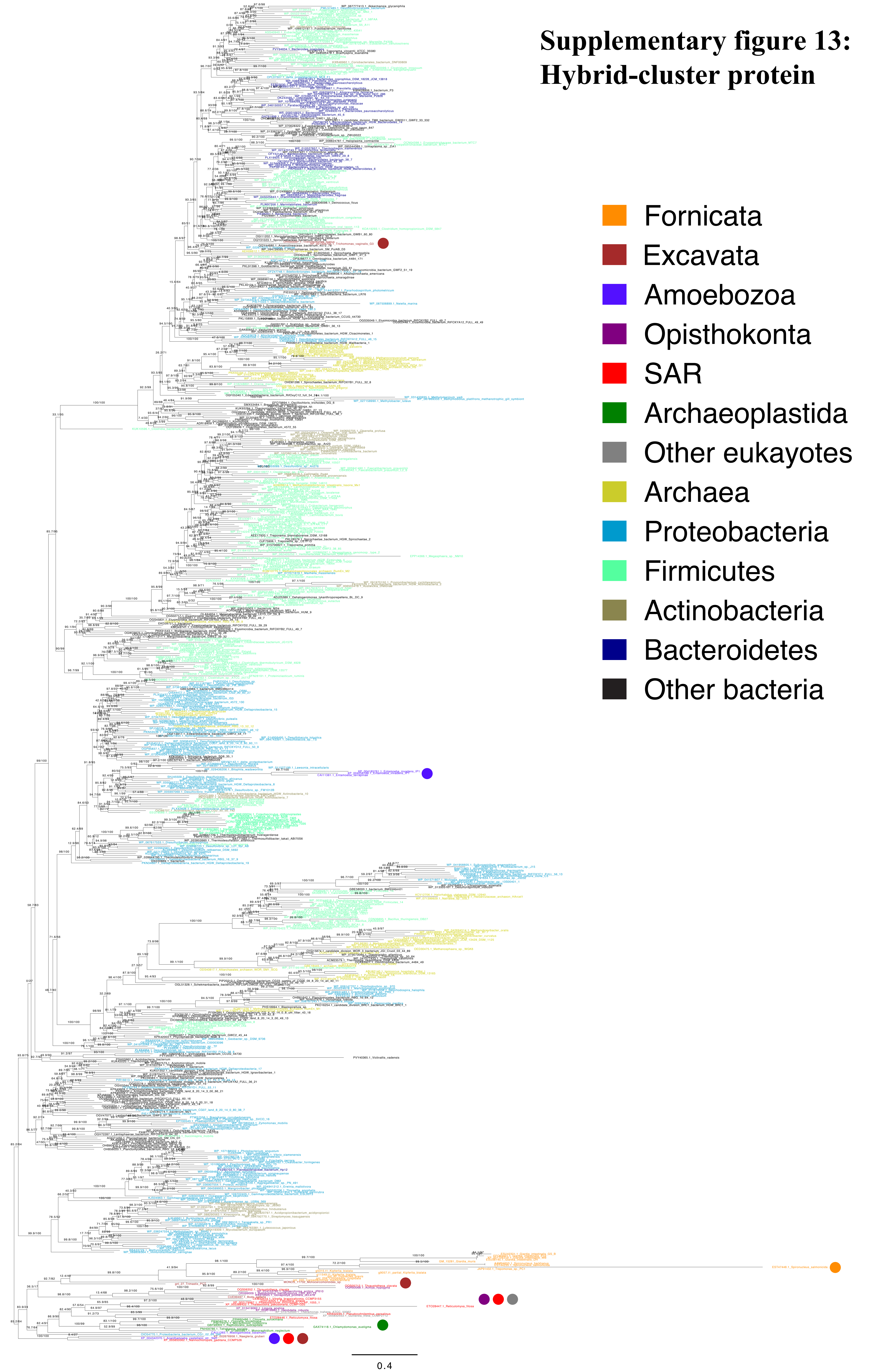

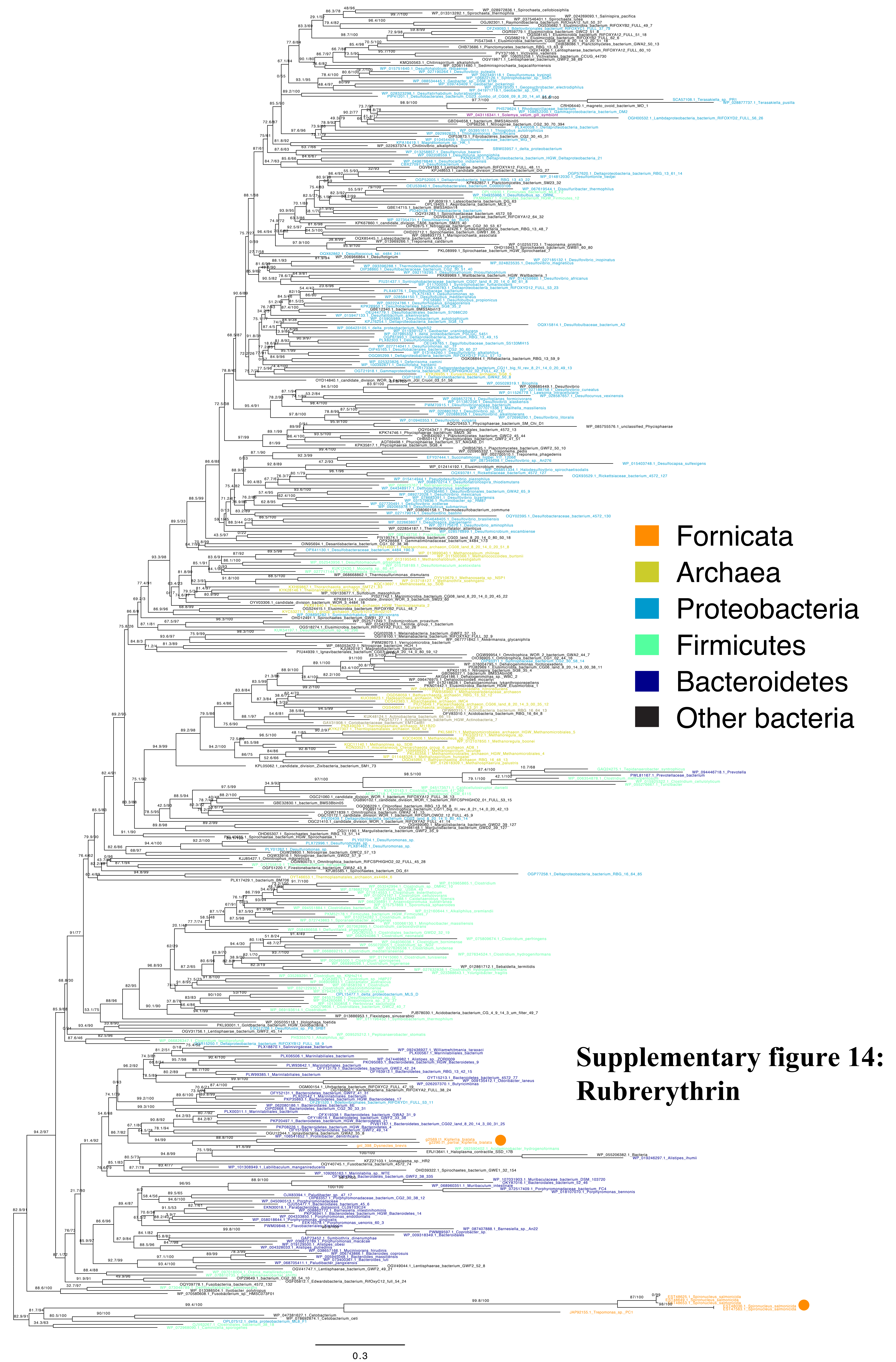

Supplementary figure 14: Rubrerythrin

Supplementary figure 15: NADH oxidase *K. bialata*

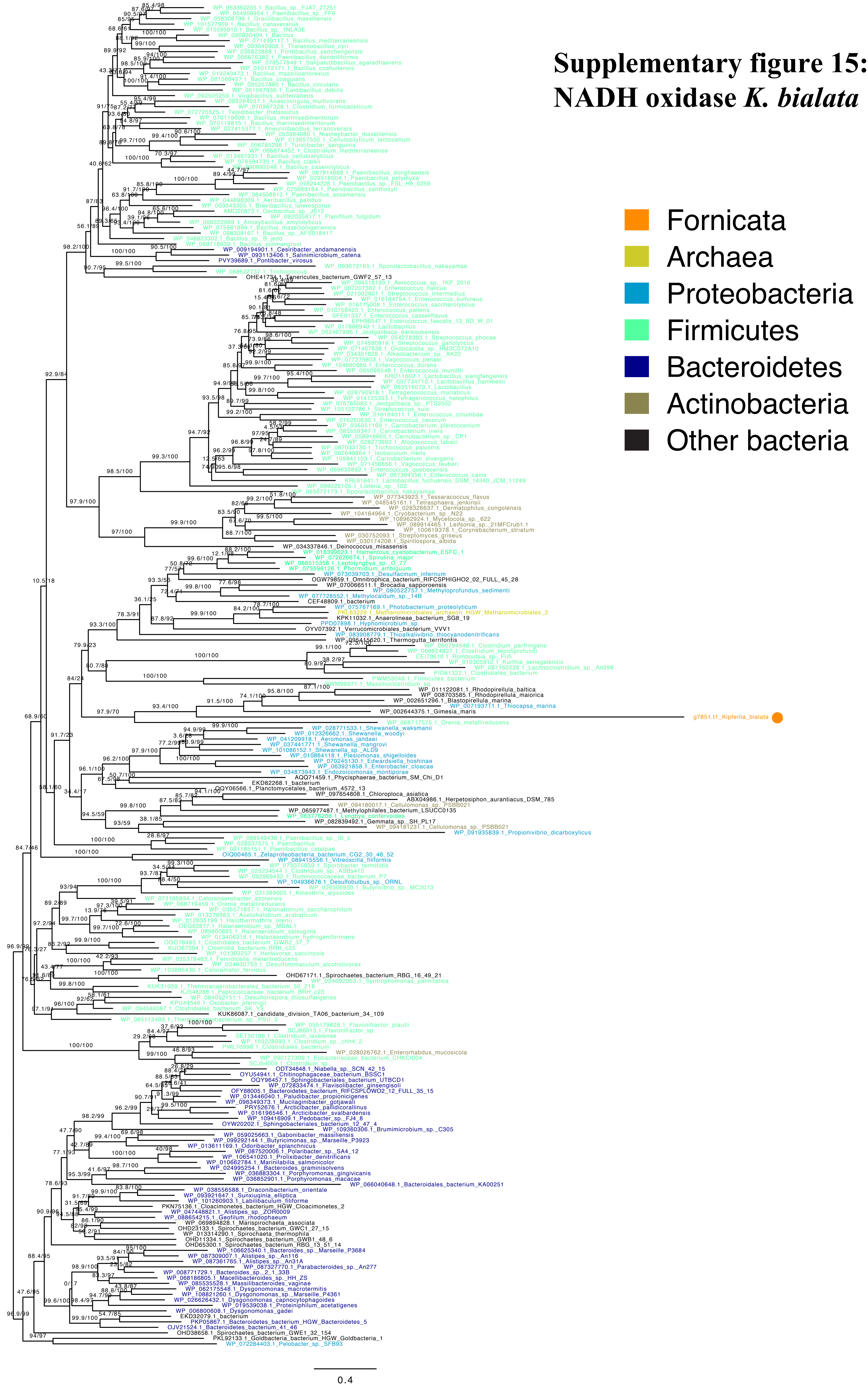

Supplementary figure 16: NADH oxidase diplomonads

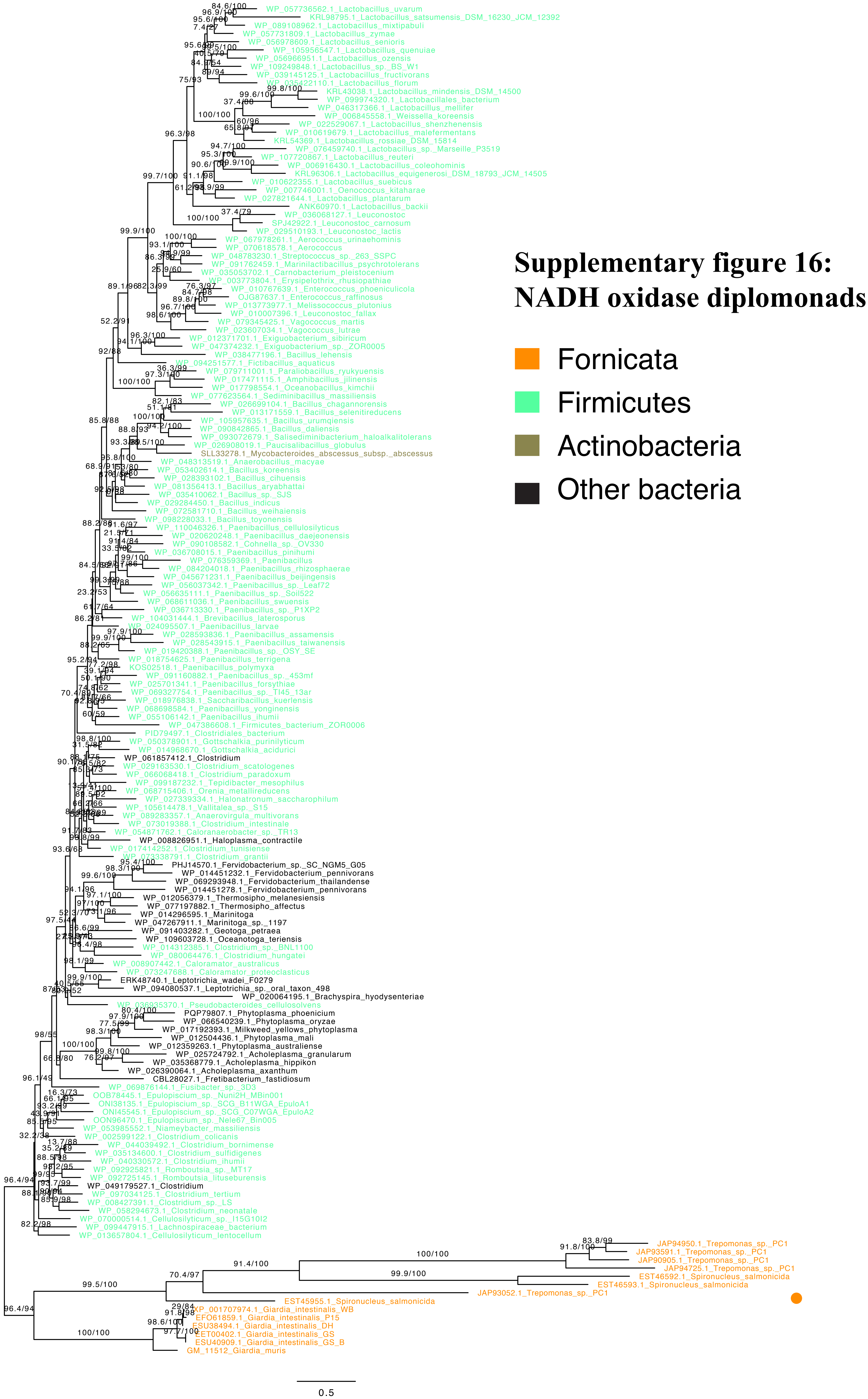

Supplementary figure 17: A-type flavoprotein

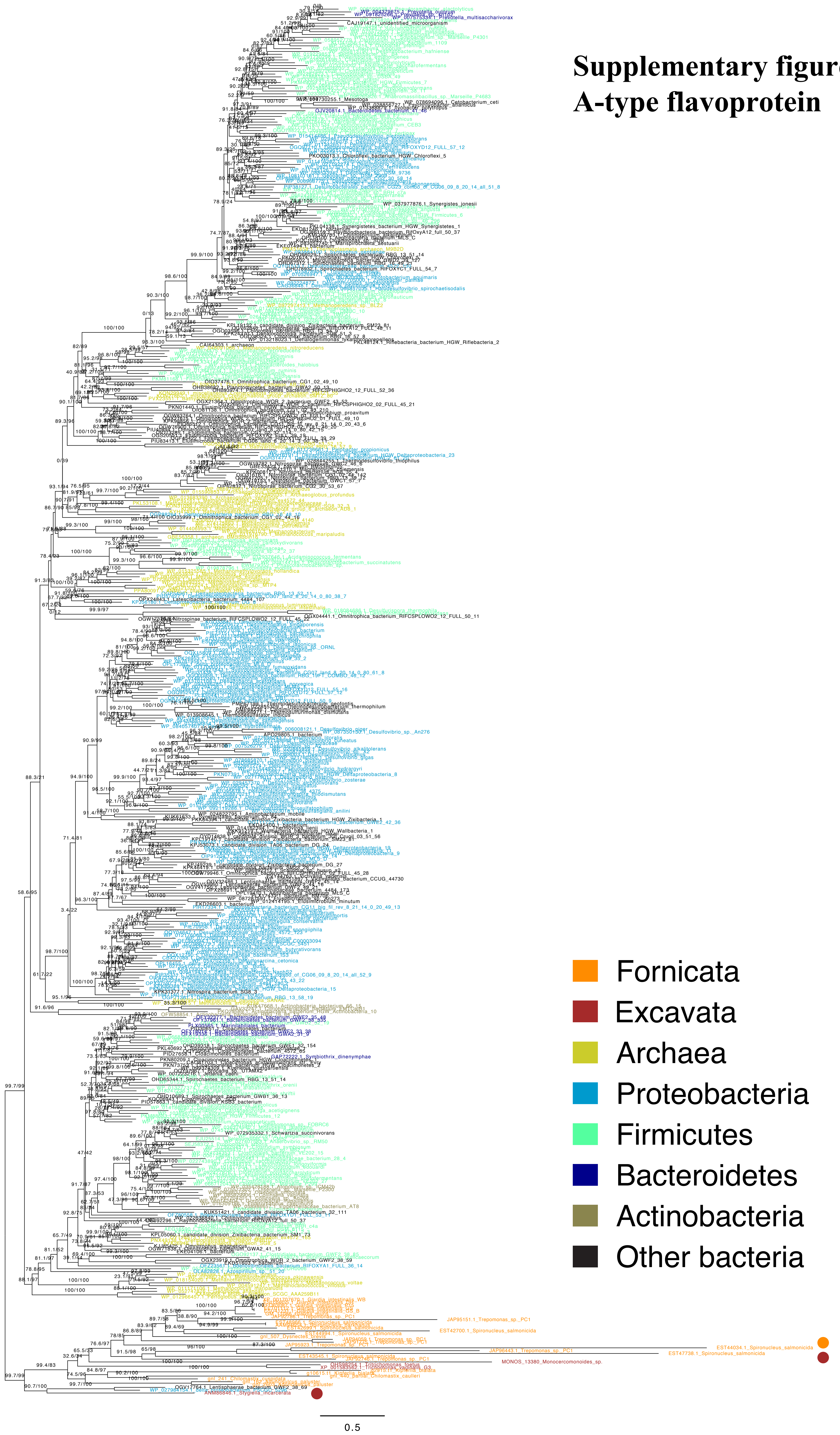

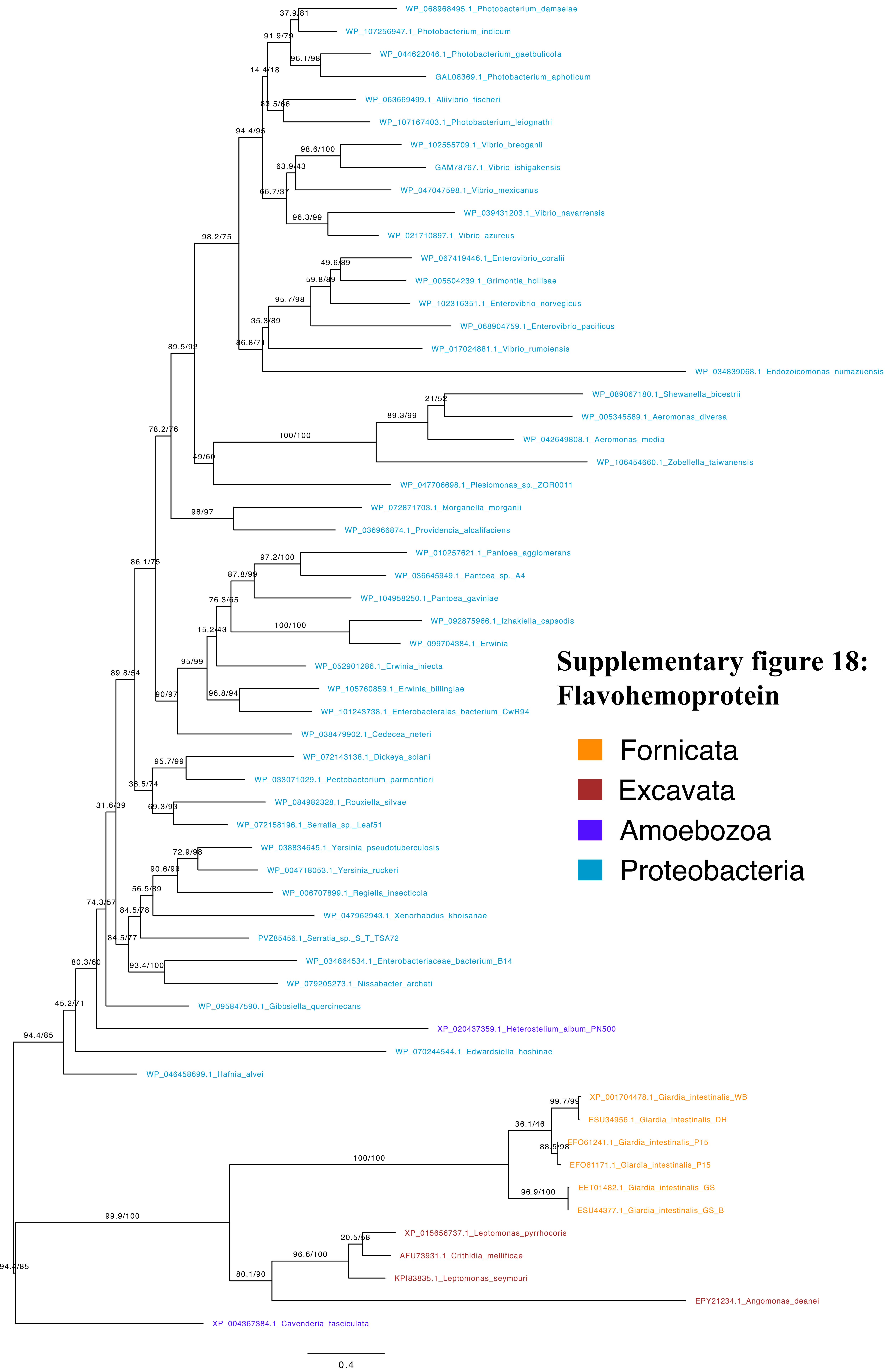

WP_068968495.1_Photobacterium_damselae
WP_107256947.1_Photobacterium_indicum
WP_044622046.1_Photobacterium_gaetbulicola
GAL08369.1_Photobacterium_aphoticum
WP_063669499.1_Aliivibrio_fischeri
WP_107167403.1_Photobacterium_leiognathi
WP_102555709.1_Vibrio_breoganii
GAM78767.1_Vibrio_ishigakensis
WP_047047598.1_Vibrio_mexicanus
WP_039431203.1_Vibrio_navarrensis
WP_021710897.1_Vibrio_azureus
WP_067419446.1_Enterovibrio_coralii
WP_005504239.1_Grimontia_hollisae
WP_102316351.1_Enterovibrio_norvegicus
WP_068904759.1_Enterovibrio_pacificus
WP_017024881.1_Vibrio_rumoiensis
WP_034839068.1_Endozoicomonas_numazuensis
WP_089067180.1_Shewanella_bicestrii
WP_005345589.1_Aeromonas_diversa
WP_042649808.1_Aeromonas_media
WP_106454660.1_Zobellella_taiwanensis
WP_047706698.1_Plesiomonas_sp._ZOR0011
WP_072871703.1_Morganella_morganii
WP_036966874.1_Providencia_alcalifaciens
WP_010257621.1_Pantoea_agglomerans
WP_036645949.1_Pantoea_sp._A4
WP_104958250.1_Pantoea_gaviniae
WP_092875966.1_Izhakiella_capsodis
WP_099704384.1_Erwinia
WP_052901286.1_Erwinia_iniecta
WP_105760859.1_Erwinia_billingiae
WP_101243738.1_Enterobacterales_bacterium_CwR94
WP_038479902.1_Cedecea_neteri
WP_072143138.1_Dickeya_solani
WP_033071029.1_Pectobacterium_parmentieri
WP_084982328.1_Rouxiella_silvae
WP_072158196.1_Serratia_sp._Leaf51
WP_038834645.1_Yersinia_pseudotuberculosis
WP_004718053.1_Yersinia_ruckeri
WP_006707899.1_Regiella_insecticola
WP_047962943.1_Xenorhabdus_khoisanae
PVZ85456.1_Serratia_sp._S_T_TSA72
WP_034864534.1_Enterobacteriaceae_bacterium_B14
WP_079205273.1_Nissabacter_archeti
WP_095847590.1_Gibbsiella_quercinecans
XP_020437359.1_Heterostelium_album_PN500
WP_070244544.1_Edwardsiella_hoshinae
WP_046458699.1_Hafnia_alvei
XP_001704478.1_Giardia_intestinalis_WB
ESU34956.1_Giardia_intestinalis_DH
EFO61241.1_Giardia_intestinalis_P15
EFO61171.1_Giardia_intestinalis_P15
EET01482.1_Giardia_intestinalis_GS
ESU44377.1_Giardia_intestinalis_GS_B
XP_015656737.1_Leptomonas_pyrrhocoris
AFU73931.1_Crithidia_mellificae
KPI83835.1_Leptomonas_seymouri
EPY21234.1_Angomonas_deanei
XP_004367384.1_Cavenderia_fasciculata
0.4
Supplementary figure 18: Flavohemoprotein
Fornicata
Excavata
Amoebozoa
Proteobacteria

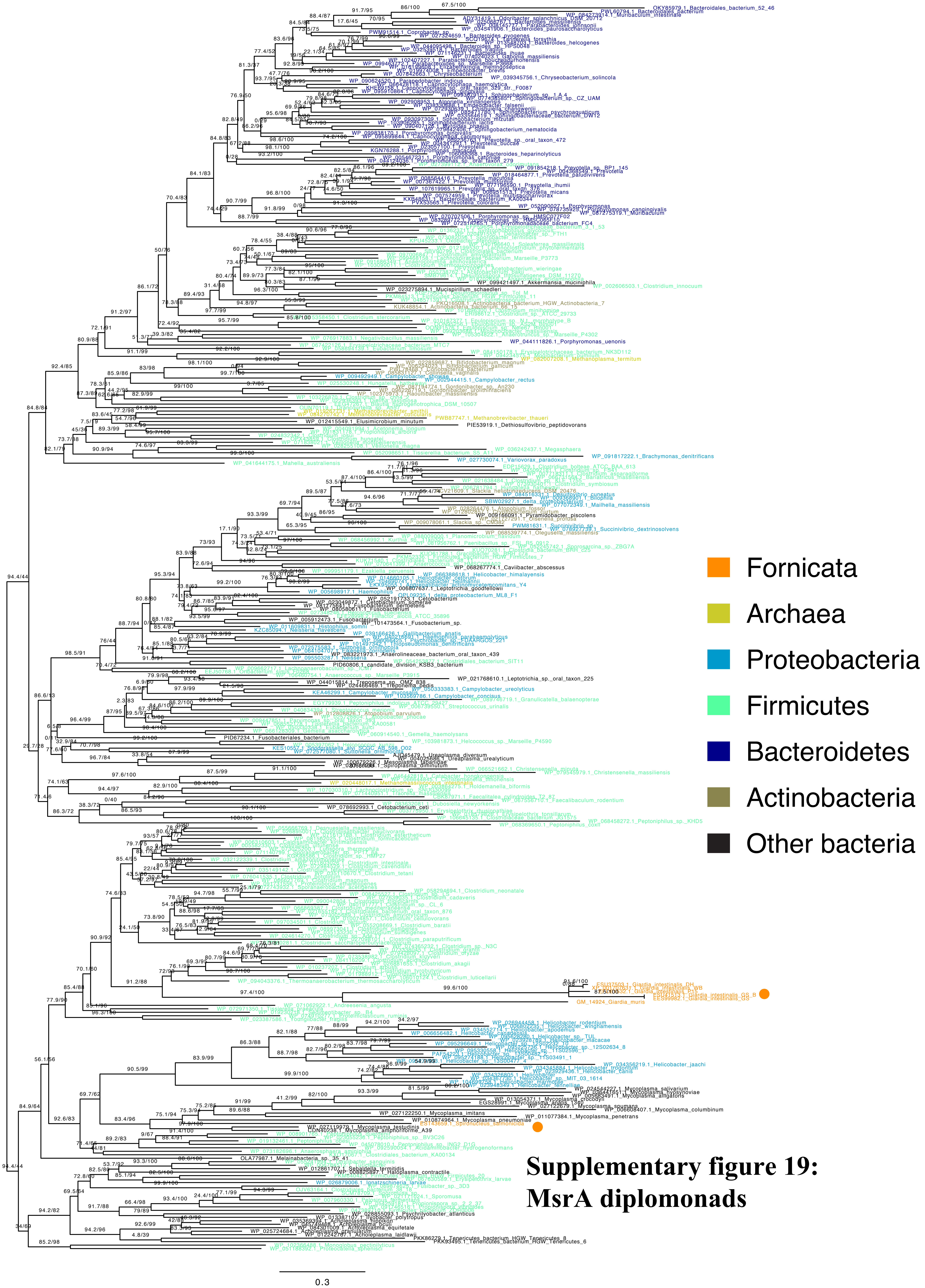

Supplementary figure 19: MsrA diplomonads

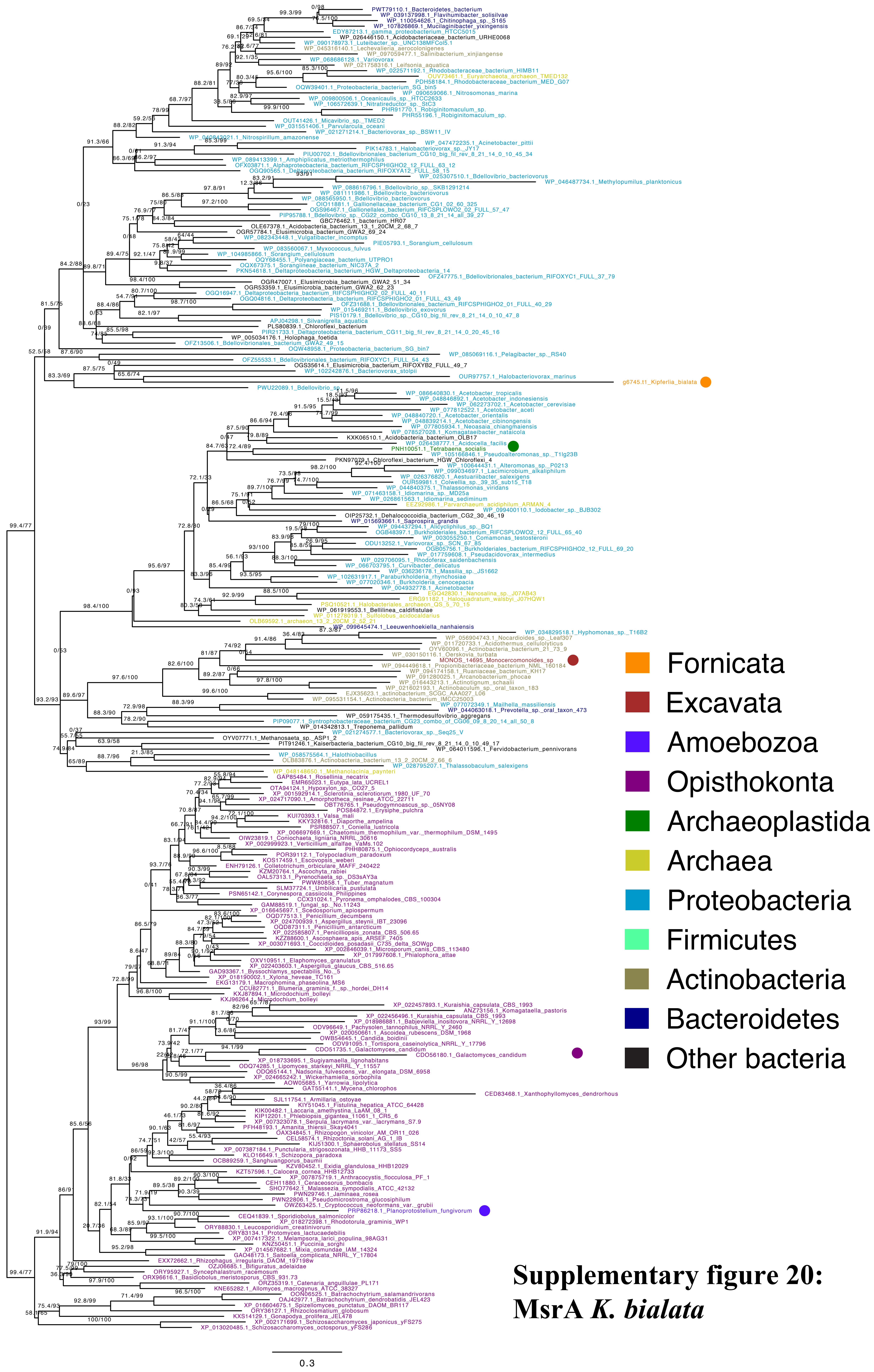

Supplementary figure 20: MsrA *K. bialata*

Supplementary figure 21: MsrB *Giardia*

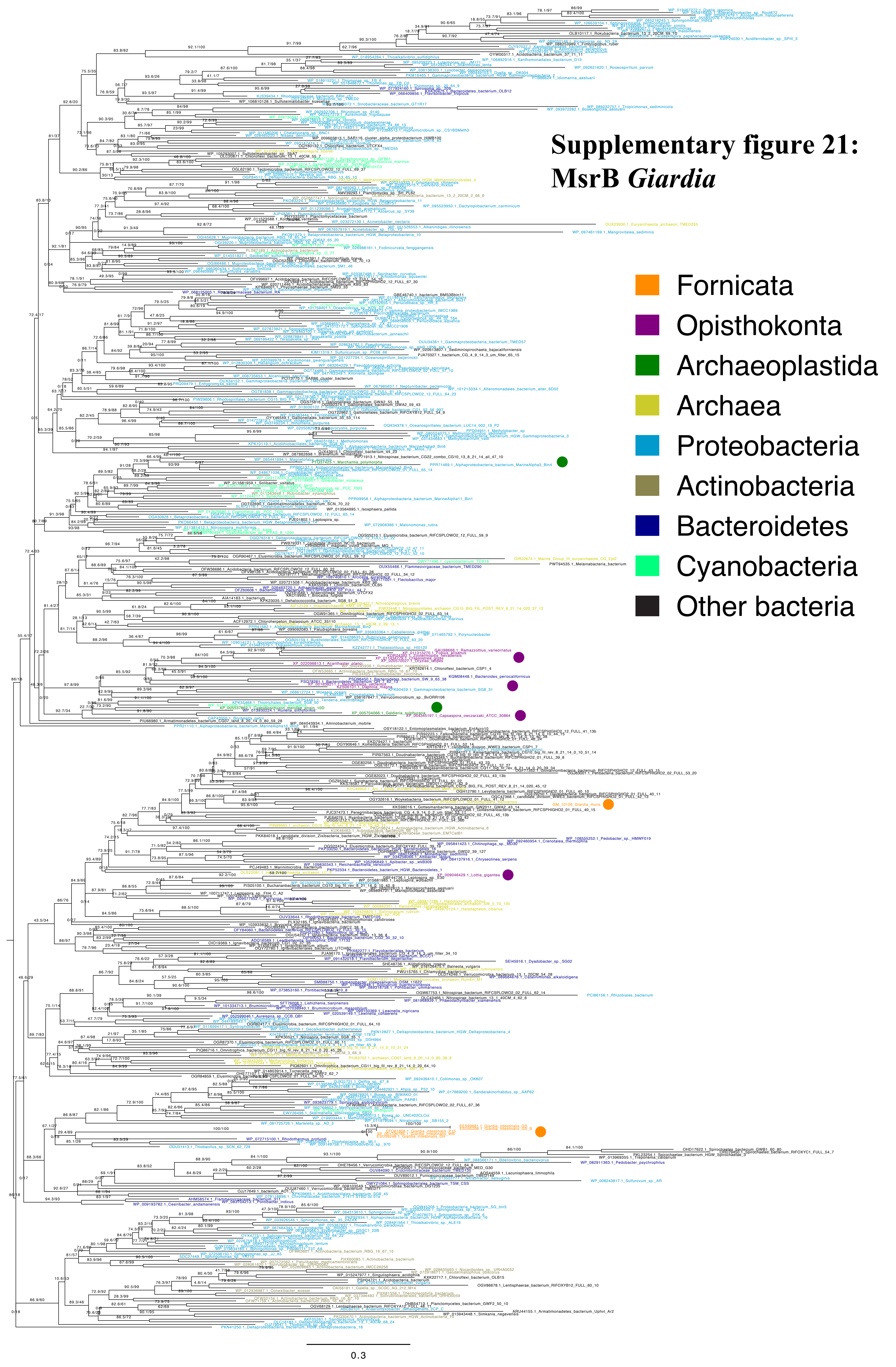

Supplementary figure 22: MsrB *K. bialata*

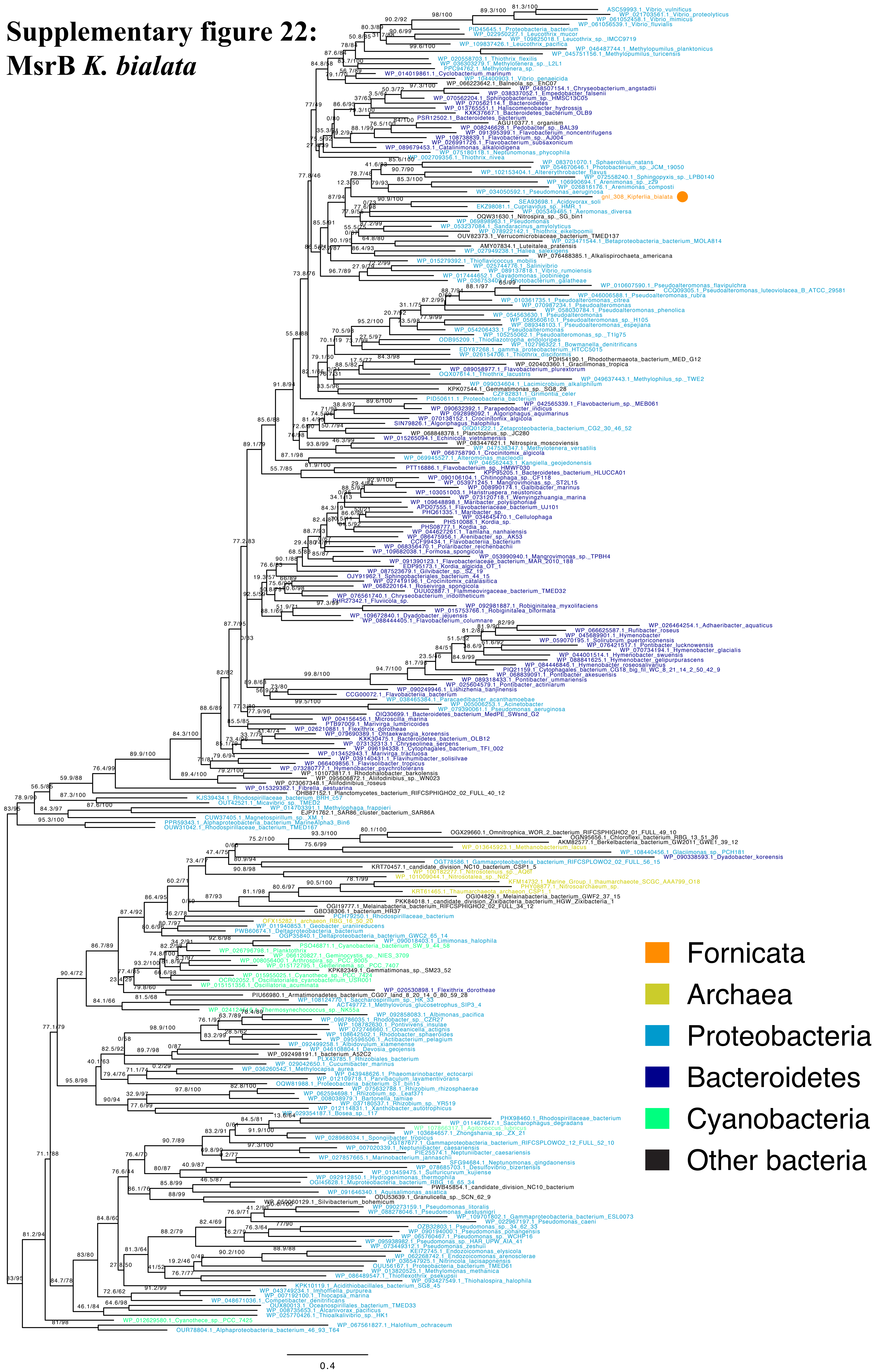

Supplementary figure 23: MsrB *S. salmonicida*

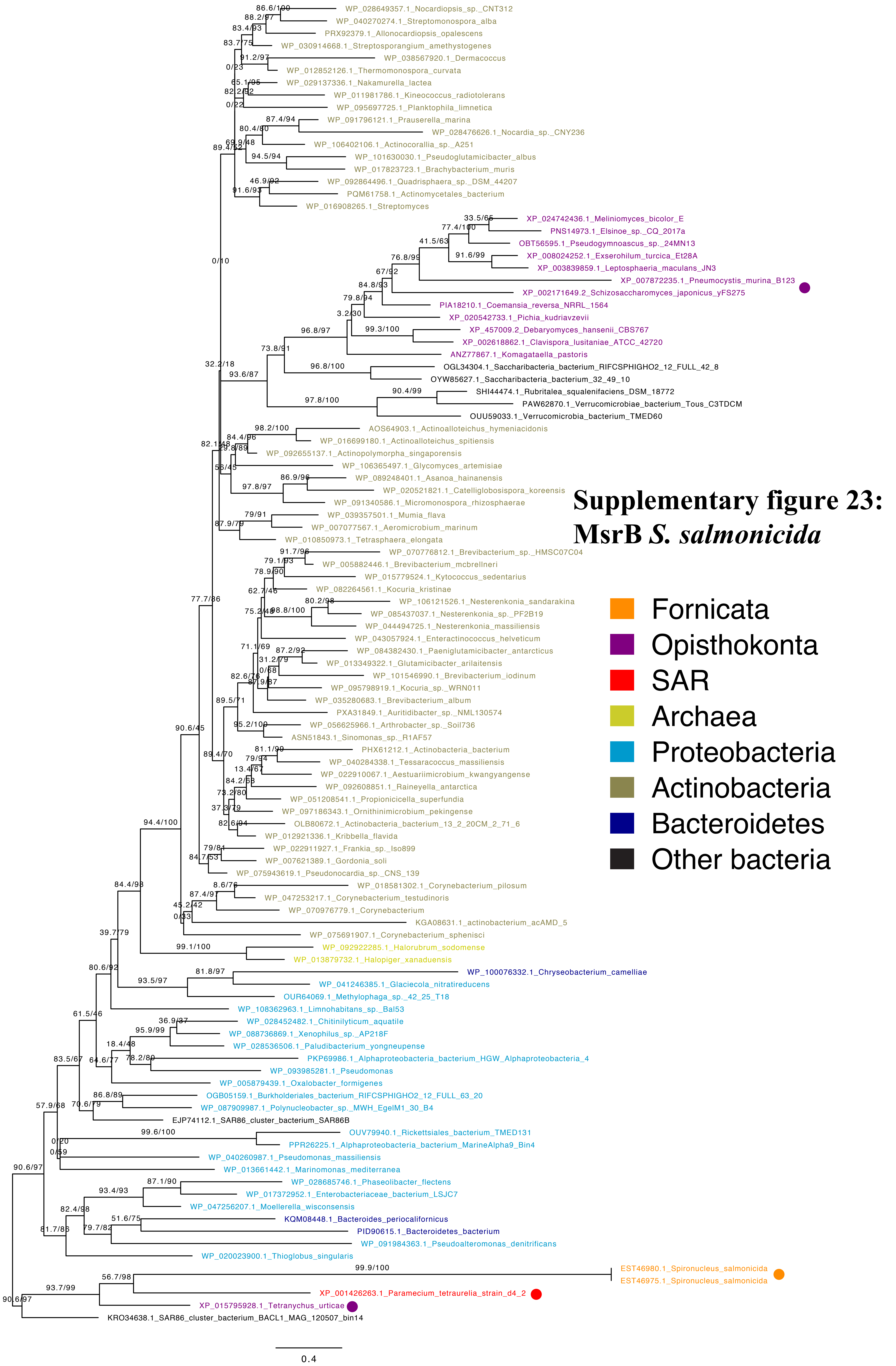

Supplementary figure 24: Phosphoserine aminotransferase

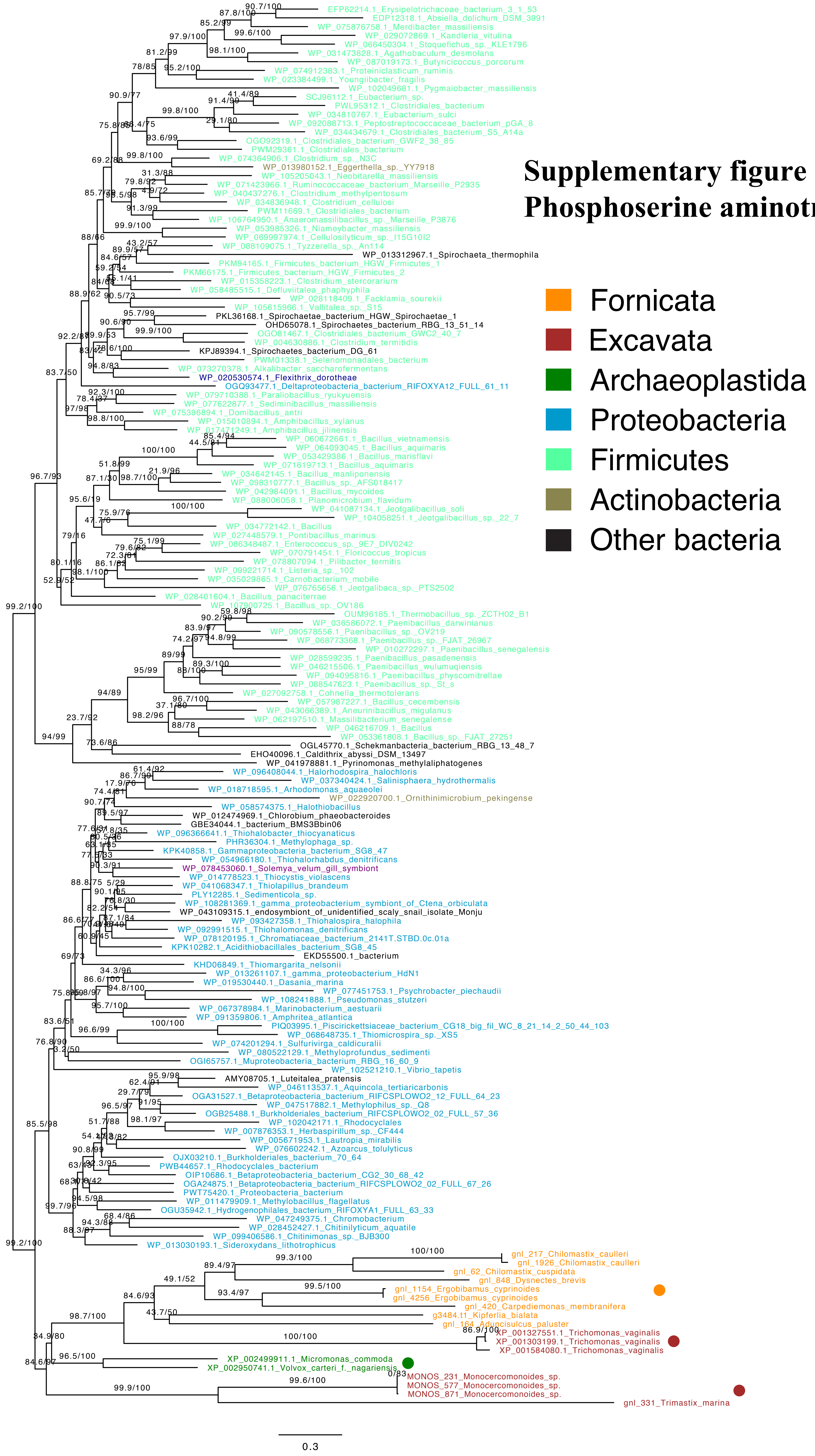

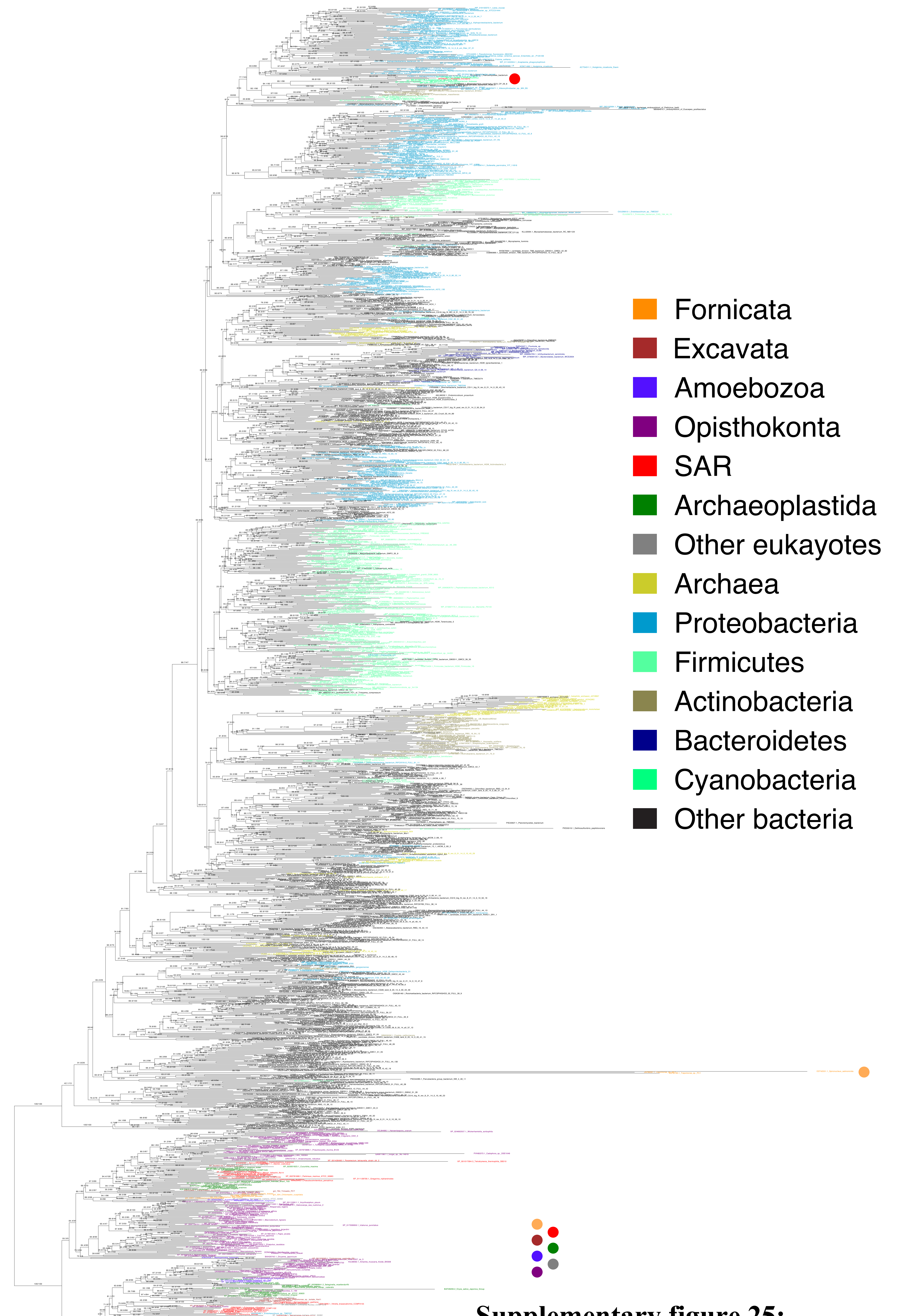

Supplementary figure 25: Serine hydroxylmethyltransferase

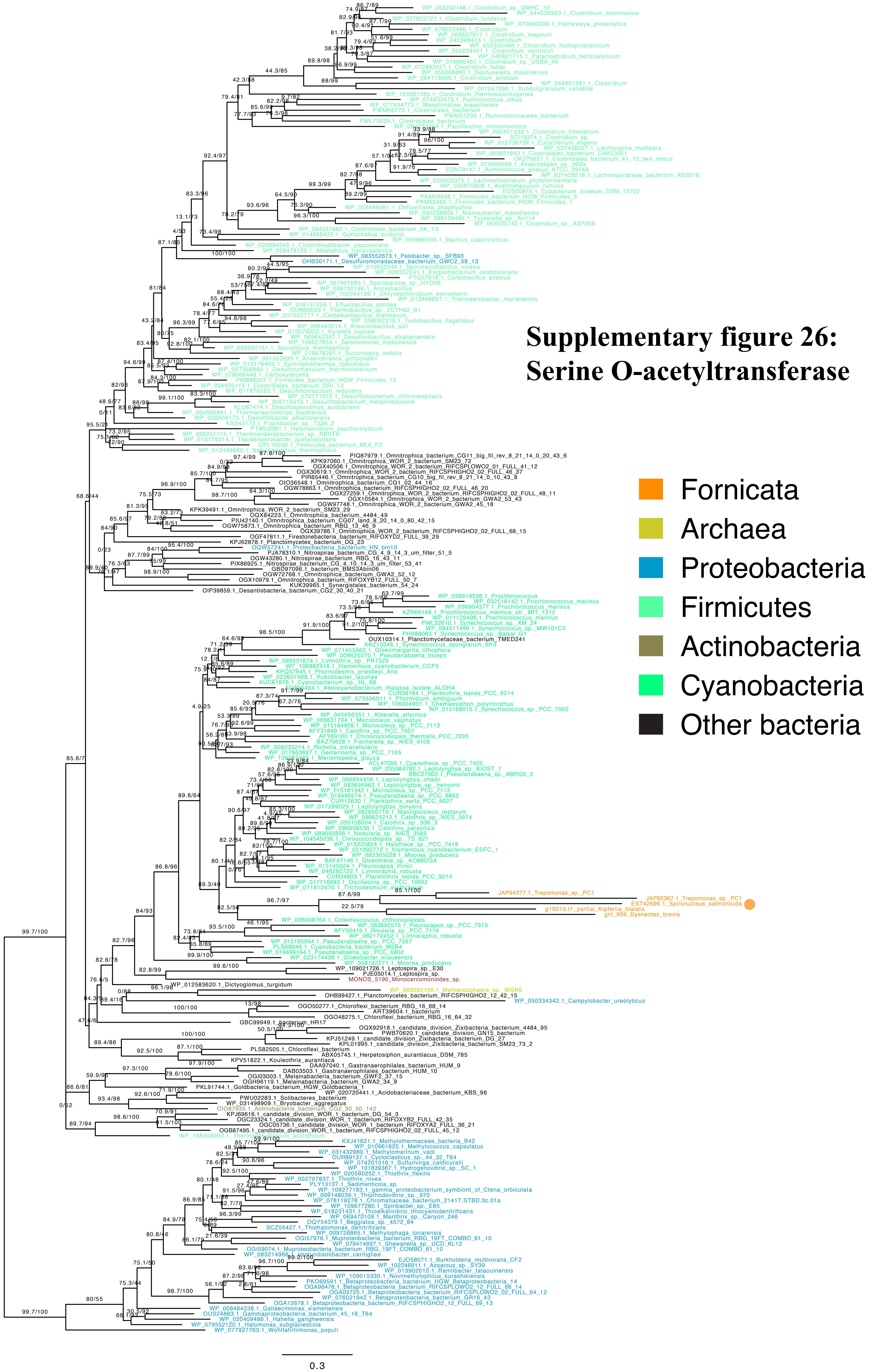

Supplementary figure 26: Serine O-acetyltransferase

Supplementary figure 27: Cysteine synthase *S. salmonicida*

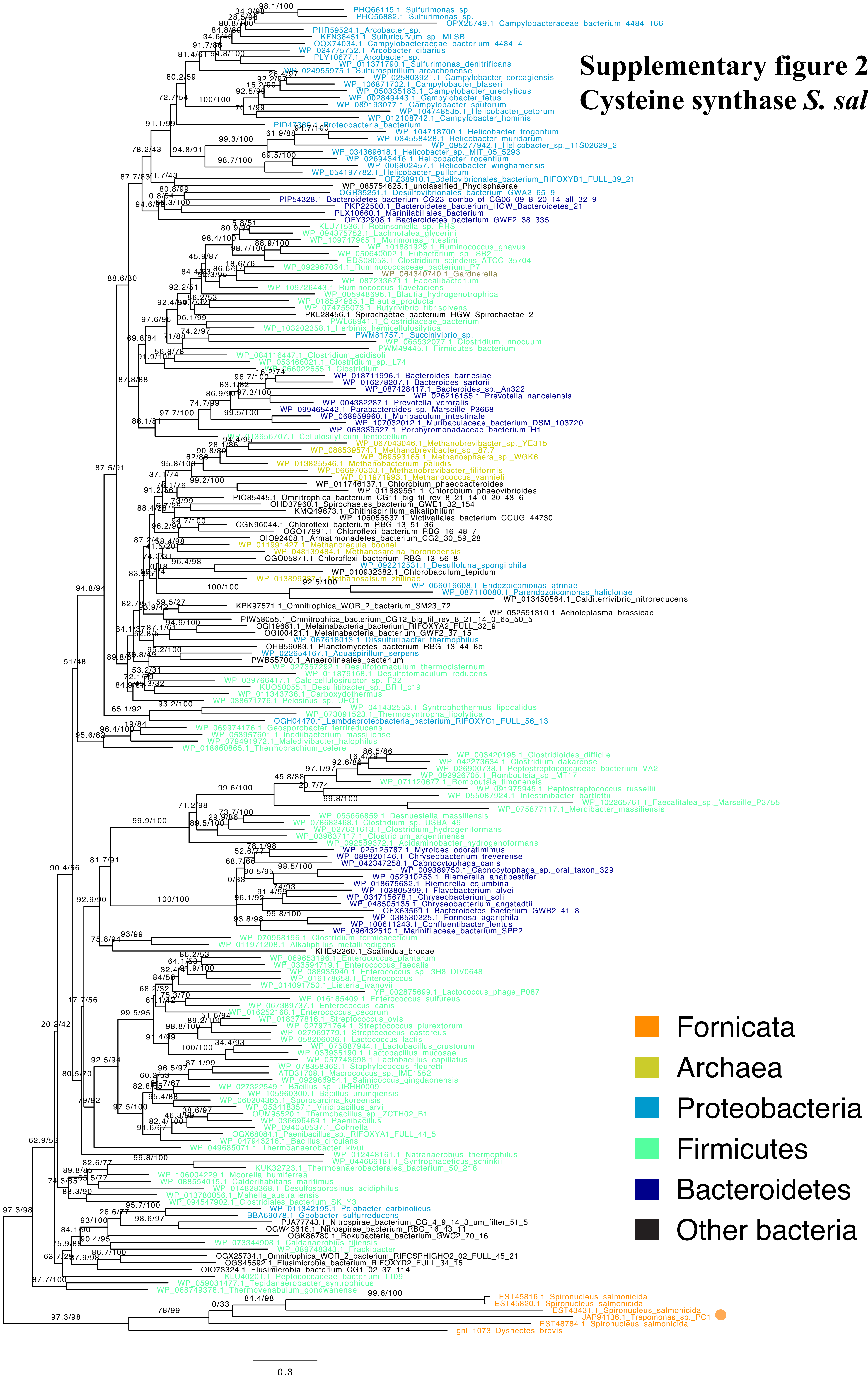

Supplementary figure 28: Cysteine synthase *K. bialata*

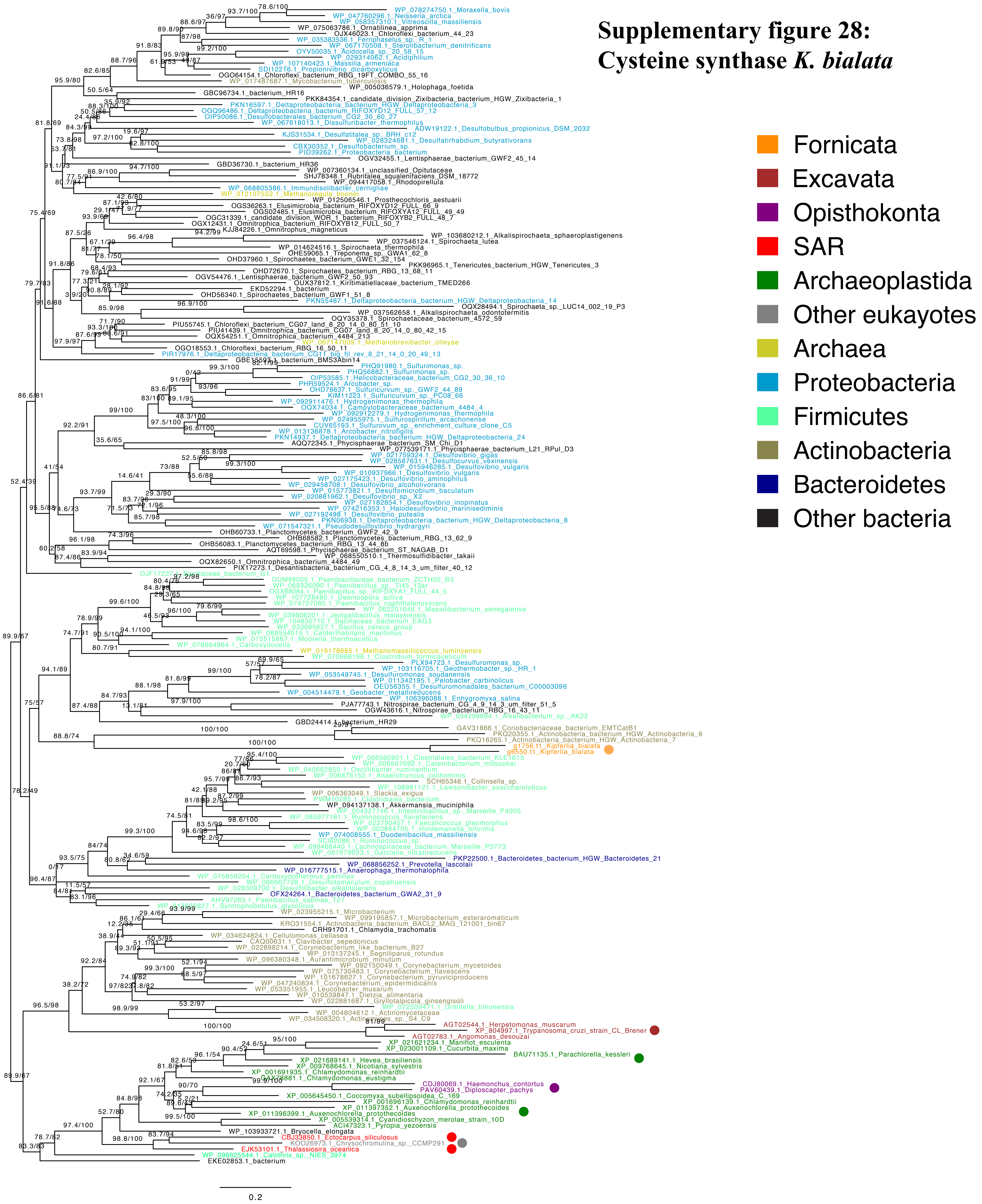

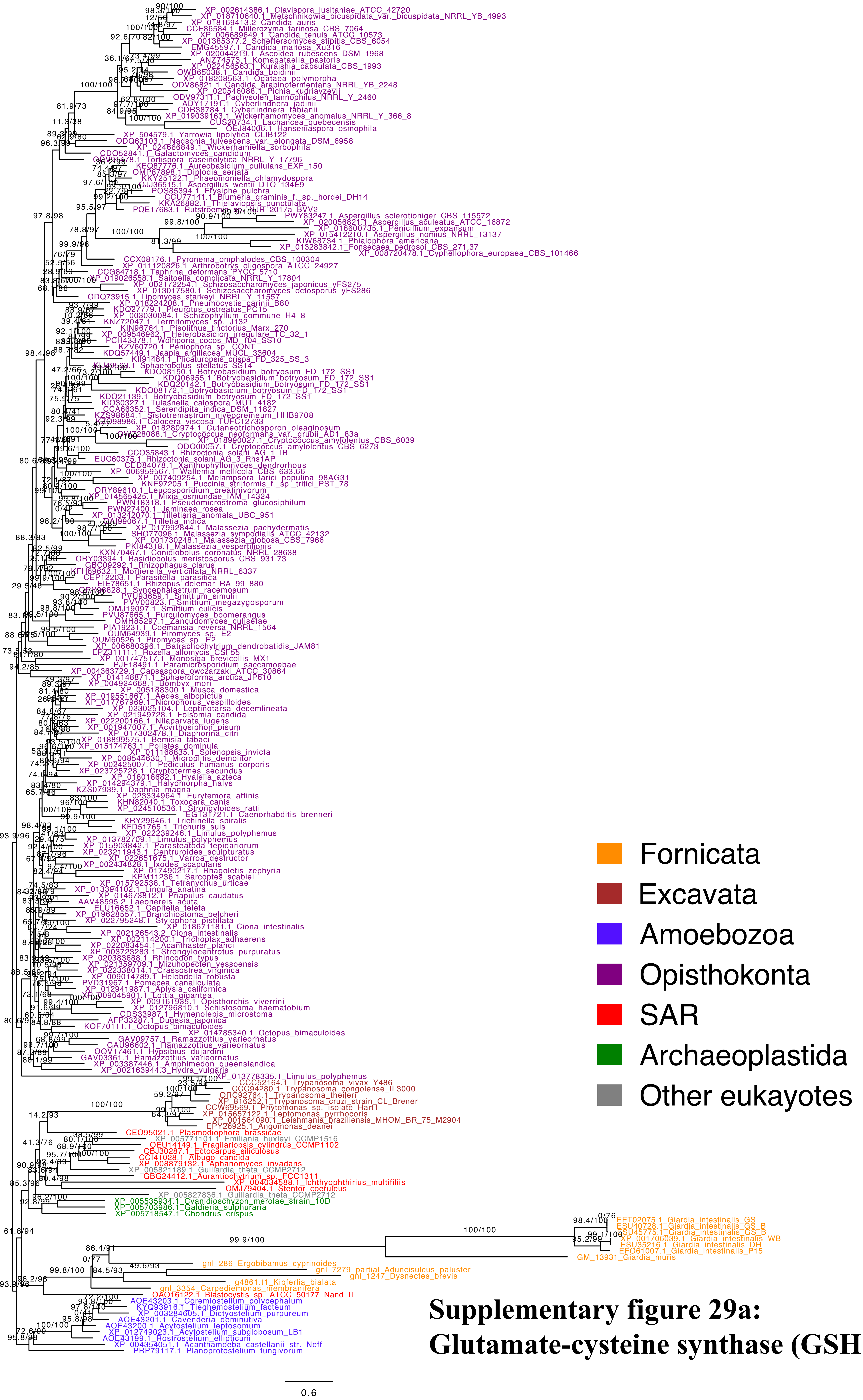

Supplementary figure 29a: Glutamate-cysteine synthase (GSH1)

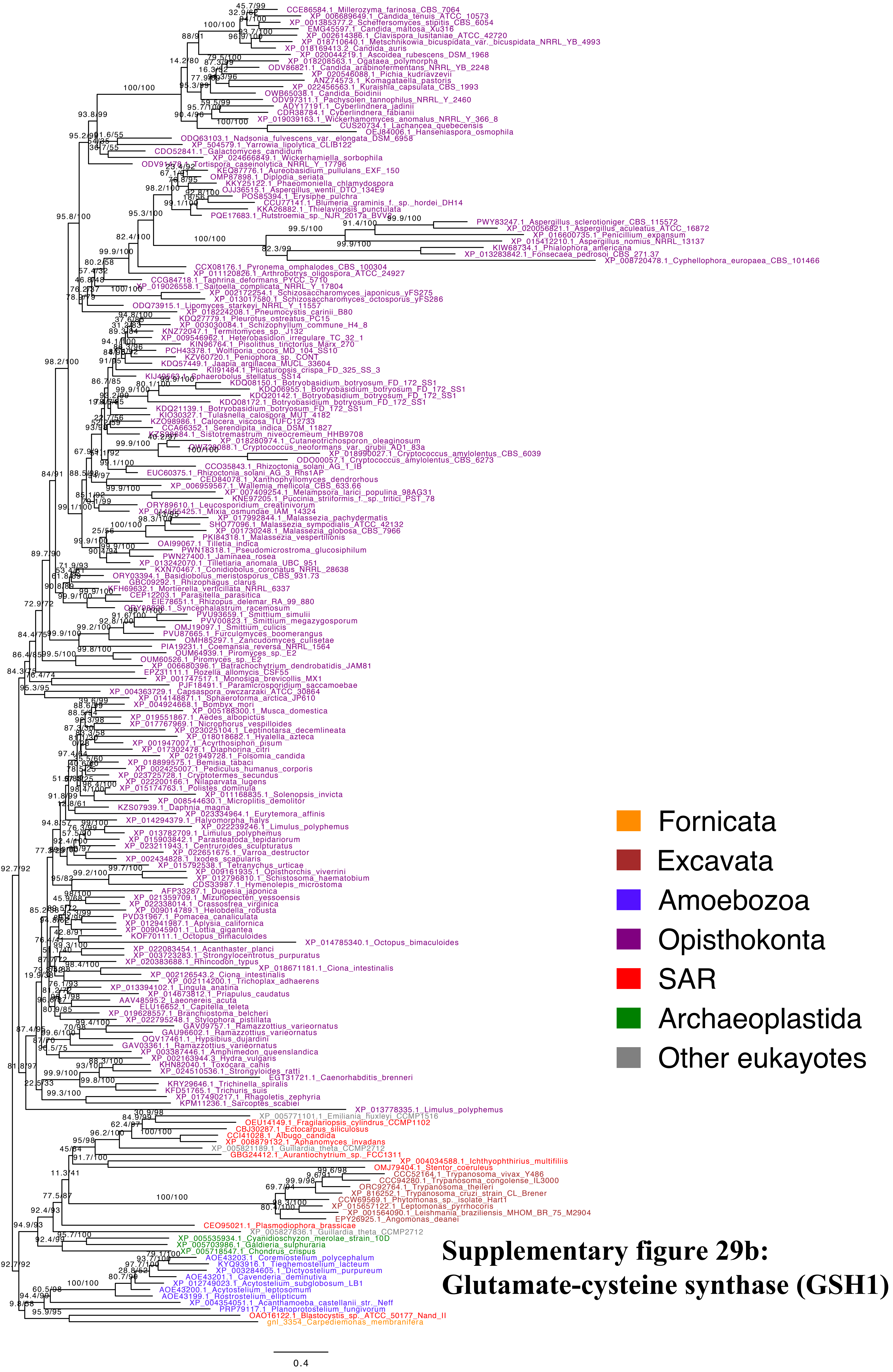

Supplementary figure 29b: Glutamate-cysteine synthase (GSH1)

Supplementary figure 30a: Glutathione synthetase (GSH2)

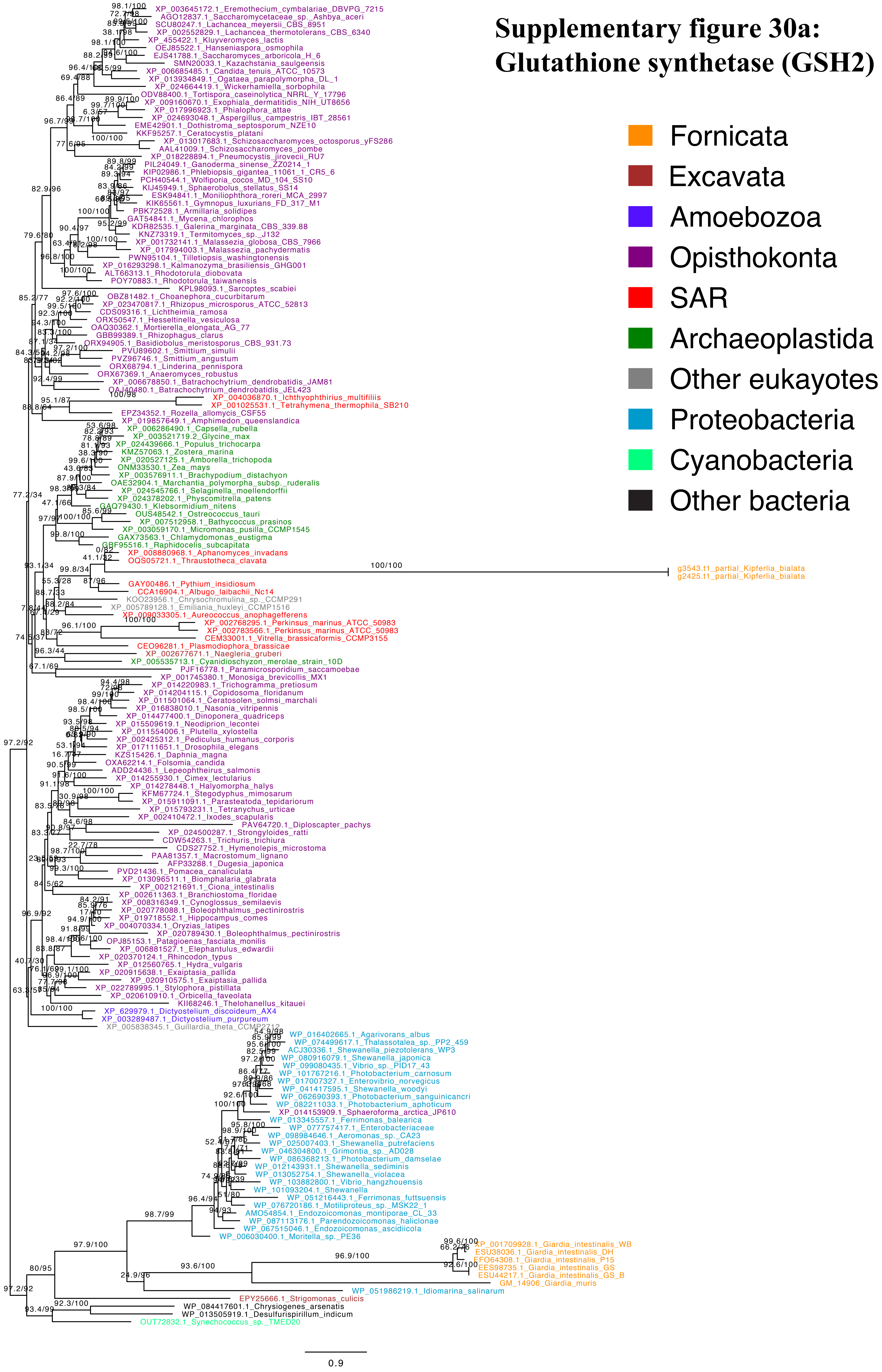

Supplementary figure 30b: Glutathione synthetase (GSH2)

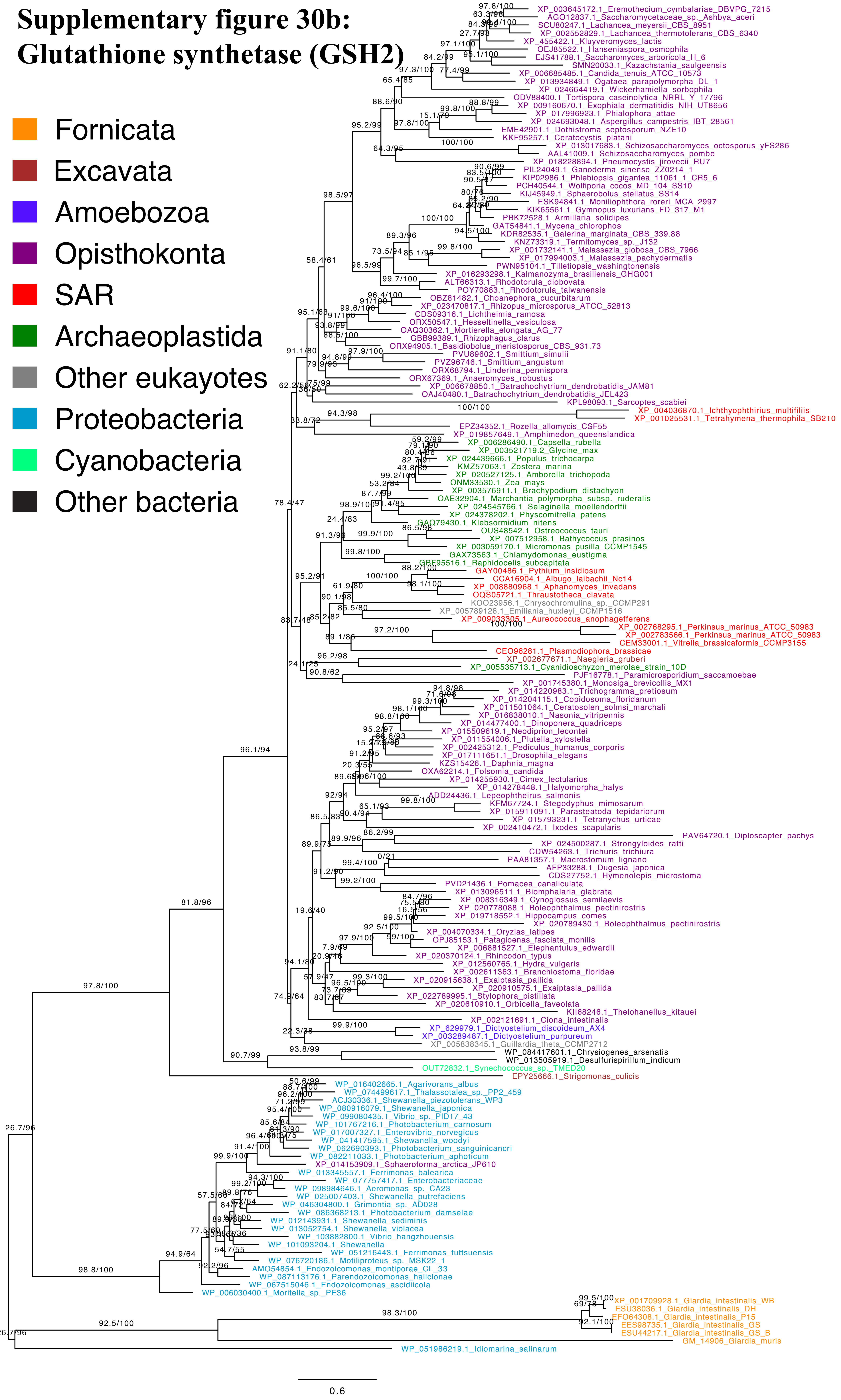

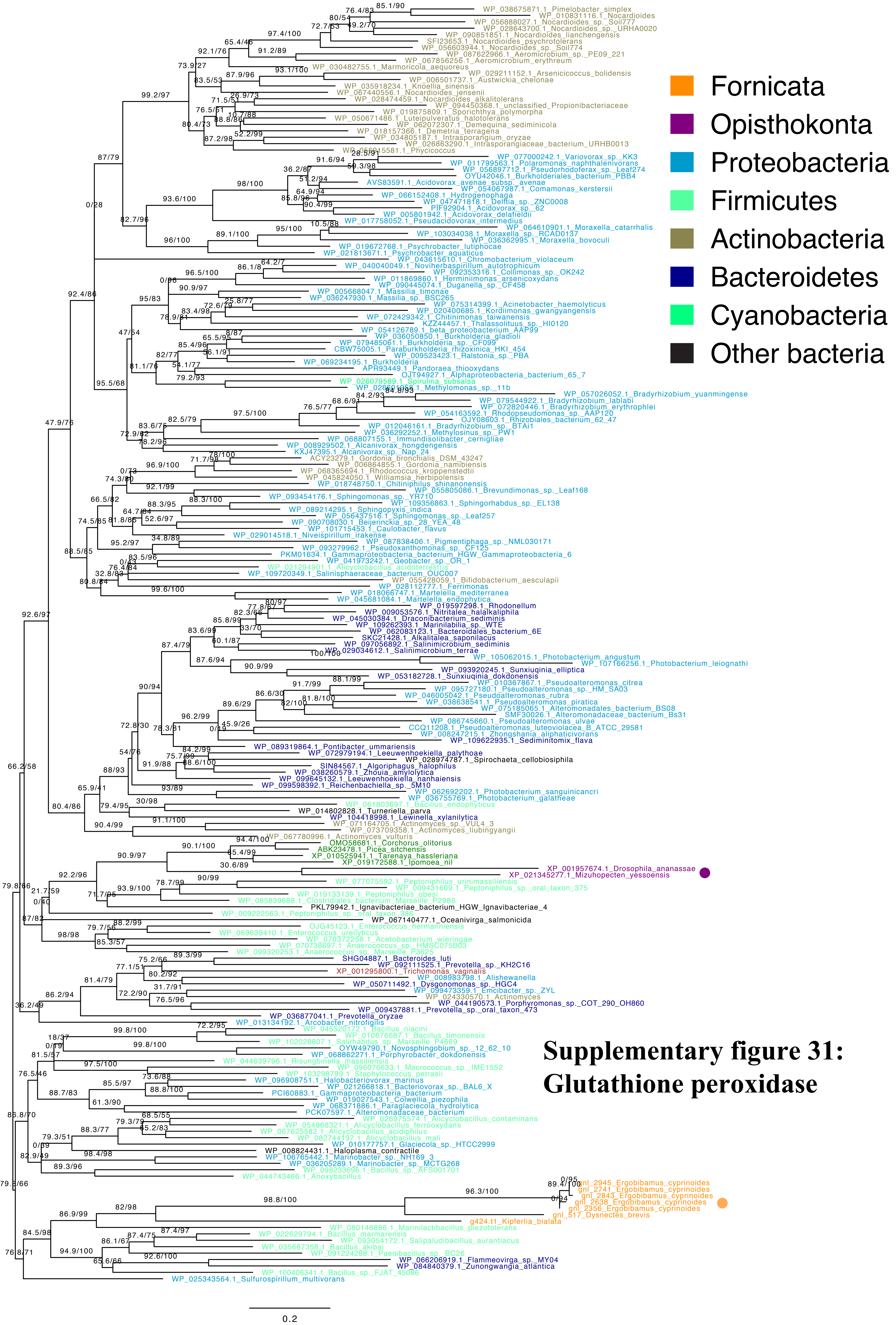

Supplementary figure 31: Glutathione peroxidase
